# Economic evaluation of direct oral anticoagulants (DOACs) versus vitamin K antagonists (VKAs) for stroke prevention in patients with atrial fibrillation: a systematic review and meta-analysis

**DOI:** 10.1136/bmjebm-2020-111634

**Published:** 2021-10-11

**Authors:** Rini Noviyani, Sitaporn Youngkong, Surakit Nathisuwan, Bhavani Shankara Bagepally, Usa Chaikledkaew, Nathorn Chaiyakunapruk, Gareth McKay, Piyamitr Sritara, John Attia, Ammarin Thakkinstian

**Affiliations:** 1 Mahidol University Health Technology Assessment (MUHTA) Graduate Program, Mahidol University, Bangkok, Thailand; 2 Department of Pharmacy, Faculty of Mathematics and Natural Sciences, Udayana University, Bali, Indonesia; 3 Social and Administrative Pharmacy Division, Department of Pharmacy, Faculty of Pharmacy, Mahidol University, Bangkok, Thailand; 4 Clinical Pharmacy Division, Department of Pharmacy, Faculty of Pharmacy, Mahidol University, Bangkok, Thailand; 5 ICMR-National Institute of Epidemiology, Chennai, India; 6 Department of Pharmacotherapy, College of Pharmacy, University of Utah, Salt Lake City, Utah, USA; 7 Centre for Public Health, School of Medicine, Dentistry and Biomedical Sciences, Queen's University, Belfast, UK; 8 Division of Cardiology, Department of Medicine, Faculty of Medicine, Ramathibodi Hospital, Mahidol University, Bangkok, Thailand; 9 School of Medicine and Public Health, Faculty of Health and Medicine, University of Newcastle, New South Wales, New South Wales, Australia; 10 Department of Clinical Epidemiology and Biostatistics, Faculty of Medicine, Ramathibodi Hospital, Mahidol University, Bangkok, Thailand

**Keywords:** health care economics and organizations, economics

## Abstract

**Objectives:**

To assess cost-effectiveness of direct oral anticoagulants (DOACs) compared with vitamin K antagonists (VKAs) for stroke prevention in atrial fibrillation (AF) by pooling incremental net benefits (INBs).

**Design:**

Systematic review and meta-analysis.

**Setting:**

We searched PubMed, Scopus and Centre for Evaluation of Value and Risks in Health Registry from inception to December 2019.

**Participants:**

Patients with AF.

**Main outcome measures:**

The INB was defined as a difference of incremental effectiveness multiplied by willing to pay threshold minus the incremental cost; a positive INB indicated favour treatment. These INBs were pooled (stratified by level of country income, perspective, time-horizon, model types) with a random-effects model if heterogeneity existed, otherwise a fixed effects model was applied. Heterogeneity was assessed using Q test and I^2^ statistic. Risk of bias was assessed using the economic evaluations bias (ECOBIAS) checklist.

**Results:**

A total of 100 eligible economic evaluation studies (224 comparisons) were included. For high-income countries (HICs) from a third-party payer (TPP) perspective, the pooled INBs for DOAC versus VKA pairs were significantly cost-effective with INBs (95% CI) of $6632 ($2961.67 to $10 303.72; I^2^=59.9%), $6353.24 ($4076.03 to $8630.45; I^2^=0%), $7664.58 ($2979.79 to $12 349.37; I^2^=0%) and $8573.07 ($1877.05 to $15 269.09; I^2^=0%) for dabigatran, apixaban, rivaroxaban and edoxaban relative to VKA, respectively but only dabigatran was significantly cost-effective from societal perspective (SP) with an INB of $11 746.96 ($2429.34 to $21 064.59; I^2^=52.4%). The pooled INBs of all comparisons for upper-middle income countries (UMICs) were not significantly cost-effective. The ECOBIAS checklist indicated that risk of bias was mostly low for most items with the exception of five items which should be less influenced on pooling INBs.

**Conclusions:**

Our meta-analysis provides comprehensive economic evidence that allows policy makers to generalise cost-effectiveness data to their local context. All DOACs may be cost-effective compared with VKA in HICs with TPP perspective. The pooling results produced moderate to high heterogeneity particularly in UMICs. Further studies are required to inform UMICs with SP.

**PROSPERO registeration number:**

CRD 42019146610.

**Summary box**

**What is already known about this subject?**

- A large number of economic evaluation studies on direct acting oral anticoagulants (DOACs) and vitamin K antagonists (VKAs) were conducted in various healthcare settings to guide health policy makers in relation to reimbursement of DOACs.
- The previous systematic reviews that compared DOACs with VKAs for stroke prevention in atrial fibrillation did not provide an overall quantitative synthesis.

**What are the new findings?**

- This is the first quantitative meta-analysis of 100 economic evaluations (that included 144 comparisons) of all four DOACs with VKAs applying pooled incremental net benefit.
- Our findings indicated that DOACs might be significantly more cost-effective than VKAs in high-income countries using a third-party payer perspective while no DOACs were more cost-effective in upper-middle income countries (UMICs), regardless of any perspective was used.
- We found that country socioeconomic status and the methodological approach used potentially influenced the cost-effectiveness of DOACs compared with VKAs.

**Summary box**

**How might it impact clinical practice in the foreseeable future?**

- While clinical efficacy and safety of DOACs over VKAs are established, these agents, at their current pricing, are cost-effective only in high -income countries but not in UMICs due partly to the lower socioeconomic status and the small number of studies available.
- Policy makers and pharmaceutical companies should together consider potential pathways to increase access to these useful agents by considering the impact of socioeconomic status on the cost-effectiveness for UMICs and potentially low-income and middle-income countries.

## Introduction

Atrial fibrillation (AF), the most common cardiac arrhythmia,^1^ is an important global health issue^2 3^ with an incidence of 596.2 cases/100 000 population in the Global Burden of Disease Study.^3^ Recent projections based on various national databases suggest that the incidence has doubled or tripled in the past decade.^4–6^ Complications of AF, particularly stroke, lead to significant morbidity and mortality.^2 3^ Disability-adjusted life years (DALYs) lost due to AF have increased almost linearly during the past 20 years, with a current global estimate of 5·98 million DALYs lost in 2017 alone.^2^

Oral anticoagulants such as vitamin K antagonists (VKAs, eg, warfarin) and direct oral anticoagulants (DOACs) are the cornerstone of stroke prevention in AF.^7^ VKAs have several limitations including the need for frequent monitoring as a consequence of numerous drug interactions.^7^ DOACs (ie, dabigatran, rivaroxaban, apixaban and edoxaban) were developed to reduce these limitations. Data from controlled trials and real-world studies suggest that DOACs are non-inferior to VKAs and have some advantages^8 9^ which has led to their recommendation over VKAs in the AF guidelines of many developed countries.^1 10^

Multiple cost-effectiveness studies have compared DOACs with VKAs in various healthcare settings to inform health policy including five systematic reviews (SRs) of economic evaluations.^11–15^ However, none have provided an overall quantitative synthesis of their findings. Recently, SR and meta-analysis (SR–MA) of economic outcomes have been performed by converting incremental cost–effectiveness ratio (ICER) to incremental net benefit (INB), and then pooling across studies.^16–18^ The ICER, estimated by dividing incremental cost with incremental effectiveness, could be interpreted that the intervention is said to be cost-effective if it is lower than the willingness to pay (WTP) threshold. However, the ICER is controversial in some state, that is, a negative ICER may be due to a lower cost but higher effectiveness, or higher cost along with lower effectiveness of the intervention. Therefore, Crespo *et al* 16 had suggested pooling the INB across studies, defined as a difference of incremental effectiveness multiplied by WTP threshold minus the incremental cost, which could be directly interpreted, that is, a positive INB indicated favour the intervention. This quantitative synthesis requires stratification by economic factors (eg, level of country income, time horizon, perspective, economic models and so on) to minimise heterogeneity.^17 18^ This SR-MA summarises the cost-effectiveness of individual DOACs compared with VKAs for stroke prevention in patients with AF to inform policy decisions in countries with limited resources.

## Methods

This SR-MA was reported in accordance with the Preferred Reporting Items for Systematic Reviews and Meta-Analysis 2020 statement and the review protocol was registered at PROSPERO.^19^

### Data sources and search strategy

We performed a comprehensive search in PubMed, Scopus and Centre for Evaluation of Value and Risks in Health (CEVR) databases from inception to 7 December 2019, see online supplemental appendix 1. Studies were selected if they met the following criteria included patients with AF, primarily/secondarily aimed to compare VKAs (ie, warfarin or acenocoumarol or phenprocoumon or coumarin) with DOACs (ie, dabigatran, apixaban, rivaroxaban and edoxaban), and reported ICER, quality-adjusted life years (QALYs) or INB. Studies were excluded if they provided insufficient data for synthesis.

10.1136/bmjebm-2020-111634.supp1 Supplementary data

### Data extraction

Two investigators (RN and BSB) independently extracted data. Disagreement was resolved in consultation with senior authors (SY and AT). Extracted data included study characteristics, study population, interventions, economic data (ie, perspective, WTP threshold or gross domestic product estimates from the World Bank according to the study year, time-horizon, currency, economic model) and findings. In addition, data for pooling were also extracted including mean cost, incremental cost, clinical effectiveness, incremental effectiveness and ICERs together with SE, or 95% CI. Incremental costs and effectiveness were also extracted from the cost-effective plane using Web-Plot-Digitizer software V.4.2.^20 21^

### Risk of bias

We assessed risk of bias for included studies using the economic evaluations bias (ECOBIAS) checklist.^22^ The first part evaluated the overall bias which consisted of the following 11 items: narrow perspective, inefficient comparator, cost measurement omission, intermittent data collection, invalid valuation, ordinal ICER, double-counting, inappropriate discounting, limited sensitivity analysis, sponsor and reporting/dissemination. The second part specifically evaluated risk of bias of the model specifications in economic evaluations consisting of three subdomains, that is, structure of the model (four items), data (six items) and consistency (one item). Each item was graded as yes, no, partly, unclear or not applicable, where yes and no referred to high and low risk of bias, respectively.

### Data analysis

The primary outcome of interest was INB. Economic data were harmonised by converting all currency data using purchasing power parity for the year 2019.^23^ In addition, different scenarios were applied to estimate INB and its variance based on the methods suggested by Crespo *et al* 16 (as follows: INB=K×ΔE−ΔC , or INB=ΔE×(K−ICER) where K is the WTP threshold, ΔC the incremental cost, ΔE the incremental effectiveness, ICER the incremental cost and incremental effectiveness ratio), and our expanded methods are published previously,^17 18^ see online supplemental appendix 2. A positive INB indicated favouring treatment (ie, intervention is cost-effective), whereas a negative INB indicated favouring comparator (ie, intervention is not cost-effective).^16 24 25^ Heterogeneity was assessed using the Cochrane-Q test and I^2^ statistic and considered present if I^2^ ≥25% or if the p value was <0.1. The INBs were pooled across studies, stratified by country income (classified by the World Bank),^20^ time-horizon, economic model and perspective, using a random-effects model (Der Simonian and Laird method) if heterogeneity was present, or an inverse-variance model if not.^26^

Meta-regression, sensitivity or subgroup analyses were undertaken to explore sources of heterogeneity such as discount rate, WTP threshold, data source and funding source. Publication bias was assessed using Egger’s test and funnel plots where number of studies/comparisons was 10 or more. Where a funnel plot was asymmetrical, a contour-enhanced funnel plot was constructed to assess if the asymmetry was due to missing studies or heterogeneity. All analyses were performed using STATA V.16. A two-sided p<0.05 was considered statistically significant except for heterogeneity tests, in which case p<0.10 was used.

## Results

### Study selection and characteristics

**Figure 1:**
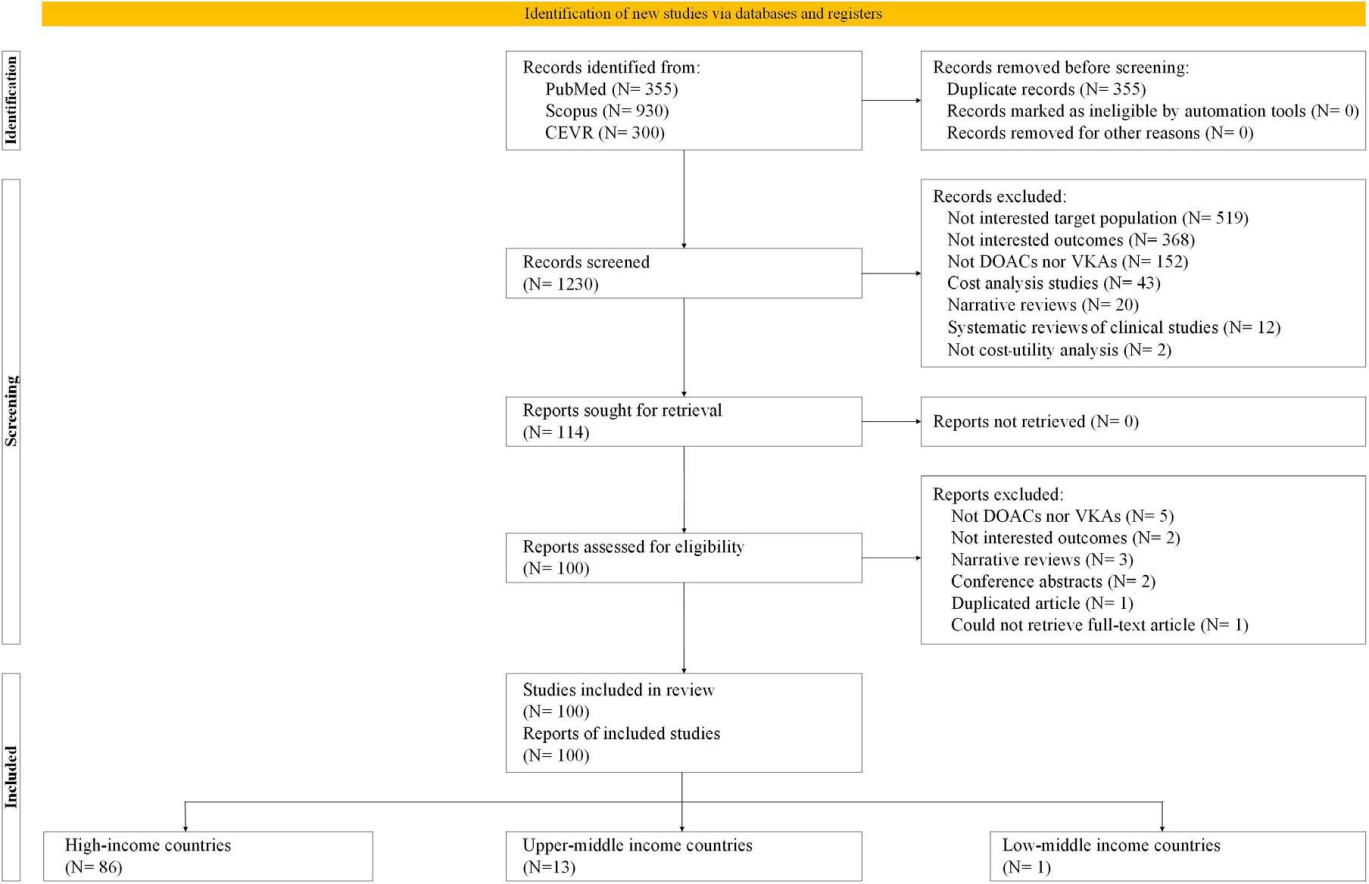
Study selection flow. DOACs, direct oral anticoagulants; VKAs, vitamin K antagonists;CEVR, Centre for Evaluation of Value and Risks in Health databases.

Of the 1585 studies identified, 100 met the inclusion criteria. List of 14 excluded studies along with reasons are provided in online supplemental appendix 3 eTable 3.1. Of those, 86, 13 and 1 study were conducted in high-income countries (HICs), upper-middle income countries (UMICs) and low/middle income country, respectively. Comparisons included dabigatran versus warfarin (N=49),^27–75^ apixaban versus warfarin (N=39),^28–30 32–41 43 45 51 62 65–67 69 72 73 76–92^ rivaroxaban versus warfarin (N=34)^28–30 32–38 40 41 43 45 50 51 57 62 65–69 72 73 83 93–101^ and edoxaban versus warfarin (N=16)^28 30 32 38 43 45 51 62 67 72 73 102–106^ (see figure 1).

Characteristics are summarised in table 1 and online supplemental appendix 3 eTable 3.2. Most studies used a third-party payer (TPP) perspective (N=83),^27 29–35 37–41 43 44 46–53 55–58 60–63 65 67–73 76 78 79 81–89 91–95 97 98 100 102 103 105–126^ followed by societal perspective (SP) (N=21)^28 33 36 43 54 57 59 64 72 75 77 80 96 99 101 104 113 123 125 127 128^ and patient perspective (N=4).^45 57 66 74^ Most studies used Markov models and a lifetime-horizon with discounting for both cost and outcomes. About 90% of studies stated no conflict of interest, and 56% were funded by pharmaceutical companies.

**Table 1:** General characteristics of the studies included (created by the authors)

Category	Number of studies (N=100)	Number of comparisons (n=224)
Perspective*		
Third-party payer	83	175
Societal	21	40
Patients	4	9
Model type		
Markov	96	216
Discrete event simulation	3	7
Economic evaluation alongside clinical trial	1	1
Time horizon		
Lifetime	96	217
Non-lifetime	4	7
Discount rate for cost		
Not reported	3	12
≤3%	53	112
>3%	44	100
Discount rate for utility*		
Not reported	3	11
≤3%	58	134
>3%	40	79
Clinical data source		
Published literature	81	181
Published literature and evidence synthesis	3	17
Published literature and registry database	11	18
Evidence synthesis	2	5
Registry database	3	3
Utility data source		
Published literature	93	209
Published literature and registry database	4	11
Survey	3	4
Currency year		
2008–2013	65	133
2014–2019	35	91
Cost-effectiveness threshold		
Country-specific	73	172
Gross domestic products-based	23	45
Others	4	7
Cost-effectiveness result*		
Cost-effective	84	166
Not cost-effective	24	58

*The total number of studies are more than 100 because individual studies applied multiple methods.

Clinical and utility parameters were mostly taken from published literature. Country-specific and GDP-based thresholds were used for WTP in 73^27–31 34–41 44 46–49 51 52 54 57–67 70 72 73 76–80 82–85 88 89 93 94 97 99–101 103–105 107–110 112–115 117–121 123–125 127 129^ and 23 studies,^33 43 45 50 55 56 68 69 71 74 75 81 86 87 91 95 96 98 106 116 122 126 128^ respectively. Eighty-four studies with 166 comparisons^29–32 34–41 44–50 52 54 56 57 60–64 66 67 69–71 73–85 87–89 91–94 96 97 99 100 102 104–129^ reported increased cost-effectiveness with DOACs compared with warfarin/derivatives, in contrast to the remainder (58 comparisons from 24 studies) which did not.^27 28 33–36 38 40 43 50 51 55 58 59 62 65 68 72 86 95 98 101 103 107^

### Risk of bias assessment

Across all 22 items from the ECOBIAS checklist, 17 items where more than 70% of studies were graded as low risk of bias, see online supplemental appendix 3 eTable 3.3. Therefore, risk of bias was mostly low for most items with the exception of five items including narrow perspective, double-counting, inappropriate discounting, reporting and dissemination and internal consistency. However, these biases should be less influenced on pooling INBs because they were occurred in both intervention and comparator, thus, should be cancelled out when calculation of the INB (a ratio of an incremental cost and QALYs).

### Pooling of INB

#### Dabigatran versus VKAs

**Figure 2:**
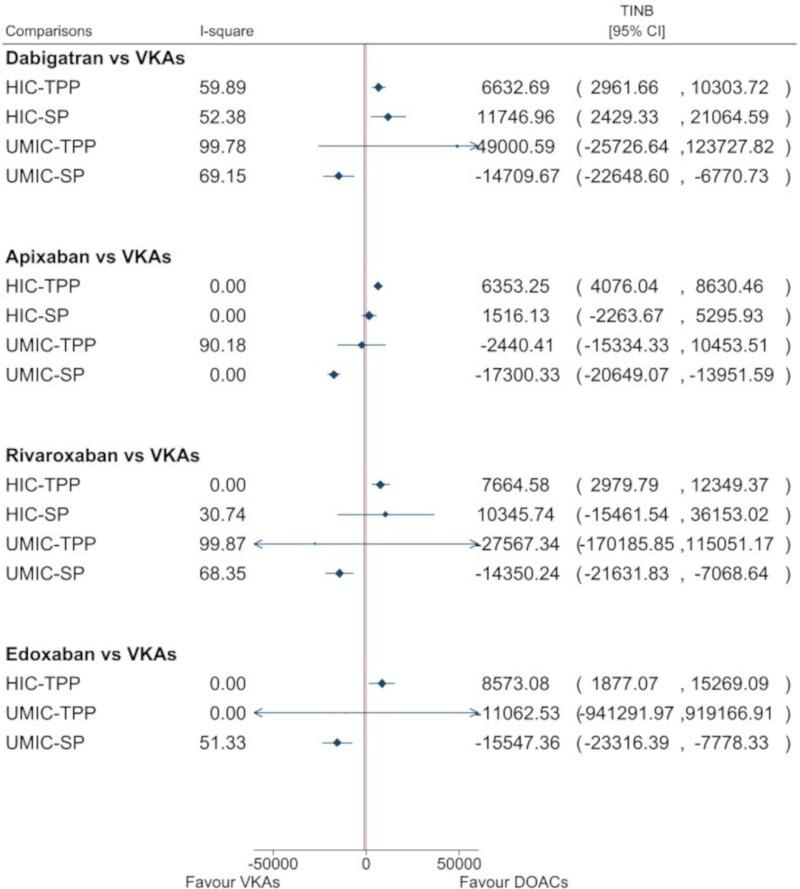
Summary of the pooled INBs of DOACs compared with VKAs classified by country income and perspectives. DOACs, direct oral anticoagulants; HICs, high-income countries; INBs, incremental net benefits; SP, societal perspective; TPP, third-party payer; UMICs, upper-middle income countries; VKAs, vitamin K antagonists.

Based on 40 studies with 48 comparisons in HICs with lifetime-horizon, the pooled INBs were $6632.70 from a TPP (95% CI $2961.67 to $10 303.72; I^2^=59.9%) and $11 746.96 from an SP (95% CI $2429.34 to $21 064.59; I^2^=52.4%). The corresponding pooled INBs in UMICs (nine studies with 13 comparisons) were $49 000.59 from a TPP (95% CI −$25 326.64 to $124 127.82; I^2^=99.8%) and −$14 709.67 from an SP (95% CI −$22 648.61 to −$6770.74; I^2^=69.2%). Dabigatran was cost-effective compared with VKAs in HICs, but not in UMICs (see figure 2, and online supplemental appendix eFigure 4.1–4.4). According to meta-regression for HICs, only funding source and WTP could partially explain heterogeneity for TPP and SP whereas heterogeneity in UMICs could not be explained (see online supplemental appendix eTable 4.1). Subgroup analysis by WTP <$50 000 and funding source from pharmaceutical companies showed that dabigatran was cost-effective compared with VKAs (online supplemental appendix 4 eFigure 4.5–4.6). Publication bias was done in the studies in HICs with TPP indicating no evidence of asymmetry, see online supplemental appendix eFigure 4.7.

#### Apixaban versus VKAs

Based on 31 studies (33 comparisons) in HICs, the pooled INBs were $6353.24 from a TPP (95% CI) $4076.03 to $8630.45; I^2^=0%) and $1516.13 from an SP (95% CI −$2263.67 to $5295.93; I^2^=0%). The corresponding pooled INBs in UMICs (eight studies with 11 comparisons) were −$2440.41 from a TPP (95% CI −$15 334.33 to $10 453.52; I^2^=90.2%), and −$17 300.33 from an SP (95% CI −$20 649.07 to −$13 951.59; I^2^=0%). Apixaban was cost-effective compared with VKAs in HICs with a TPP but not with an SP (see figure 2, and online supplemental appendix eFigures 5.1–5.4). According to meta-regression for UMICs, only discount rates for cost/utility and clinical data source could explain heterogeneity for a TPP whereas the other factors could not explain heterogeneity (see online supplemental appendix eTable 5.1, eFigure 5.5–5.8). There was no evidence of asymmetry using funnel plots and Egger’s tests for those studies in HICs with TPP, see online supplemental appendix eFigure 5.9.

#### Rivaroxaban versus VKAs

Based on 26 studies with 28 comparisons in HICs, the pooled INBs were $7664.58 from a TPP (95% CI $2979.79 to $12 349.37; I^2^=0%) and $10 345.74 from an SP (95% CI −$15 461.54, $36 153.02; I^2^=30.7%). The corresponding pooled INBs in UMICs (seven studies with 10 comparisons) were −$27 567.34 from a TPP (95% CI −$170 185.85 to $115 051.17; I^2^=99.9%), and −$14 350.24 from an SP (95% CI −$21 631.83 to −$7068.64; I^2^=68.3%). Rivaroxaban was cost-effective compared with VKAs in HICs with lifetime-horizon from TPP, but not from SP, see figure 2, and online supplemental appendix eFigure 6.1–6.4. Furthermore, rivaroxaban was significantly not cost-effective compared with VKAs in UMICs. According to meta-regression for UMICs with TPP, none of economic factors could explain heterogeneity (see online supplemental appendix eTable 6.1). There was no evidence of asymmetry for pooling INBs in HICs and TPP, see online supplemental appendix eFigure 6.5.

#### Edoxaban versus VKAs

Based on 13 studies with 15 comparisons in HICs, the pooled INBs (95% CI) were $8573.07 from a TPP (95% CI $1877.05 to $15 269.09; I^2^=0%). The pooled INBs in UMICs (three studies with five comparisons) were −$11 062.53 from a TPP (95% CI −$941 291.97 to $919 166.9; I^2^=0%) and −$15 547.36 from an SP (95% CI −$23 316.39 to −$7778.33; I^2^=51.3%). Edoxaban was cost-effective compared with VKAs from TPP only in HICs, but not cost-effective in UMICs in both TPP and SP, see figure 2, and online supplemental appendix eFigure 7.1–7.3. Source of heterogeneity could be not explored for pooling in UMICs and SP due to very small number of studies.

## Discussion

This SR-MA assessed whether DOACs were more cost-effective than VKAs for preventing stroke in patients with AF. The INBs were pooled, stratified by country income, economic models, time-horizon, as well as perspective. Data from 100 studies with 224 comparisons of DOACs to VKAs were included. The pooled INBs associated with four DOACs (ie, dabigatran, apixaban, rivaroxaban and edoxaban) from a TPP were significantly more cost-effective in HICs compared with VKAs. However, outcomes varied if the evaluation was conducted from an SP; with only dabigatran remaining cost-effective compared with VKAs. Conversely, all DOACs were not cost-effective compared with VKAs in UMICs with SP.

To our knowledge, this is the first SR-MA of cost-effectiveness that includes all four commonly used DOACs providing quantitative economic evidence. Given the variable reporting of economic outcomes, the use of INBs provides direct interpretation and supporting evidence for policy decision making. To minimise the heterogeneity across economic studies, we initially pooled INBs from similar studies based on strata including country incomes, economic model, perspectives and time-horizon. Heterogeneity was therefore reduced in studies from HICs but remained moderate to high in UMICs. This may be due to variation in the characteristics and assumptions that underlie the key model features, different reporting mechanisms, and measures of dispersion for point estimates within individual studies. As such, different approaches, data simulations and variance values were considered from similar studies in our analyses.^17 18^

Our study found that country socioeconomic status and methodological approach used potentially influenced the cost-effectiveness of DOACs versus VKAs. DOACs were cost-effective in HICs when the evaluation was conducted using Markov models and lifetime-horizon from TPP-perspective but only dabigatran was cost-effective when using SP. This paradox could be explained by the much smaller number of previous studies analysed from SP in HICs. Moreover, many of them originated from the USA where the WTP thresholds were higher than those from other HICs. Hence, even though DOACs were cost-effective in comparison to VKAs in some individual studies, once their INBs were pooled, the effect was lost.

It is noteworthy that subgroup analyses highlighted that dabigatran was significantly cost-effective compared with VKAs from TPP when WTP thresholds were less than $50 000. Therefore, policy makers in HICs should consider these conditions in their decision making especially when the SP is preferred or the WTP threshold is less than $50 000 per QALY.

Our findings confirm the individual economic evaluations in UMICs that all DOACs were less cost-effective than VKAs particularly with SP and low WTP thresholds. However, apixaban might be more cost-effective than VKAs when considered according to WTP threshold. In general, DOACs would not be the optimum choice compared with VKAs in UMICs. Many of the economic evaluations of DOACs versus VKAs for stroke prevention in patients with AF are represented by diverse methods.

### Strengths and weaknesses of the study

Our study provides comprehensive economic evidence for policy makers to assess cost-effectiveness data in their local context, considering perspectives, time horizons, discounting, sources of data and WTP thresholds. Our study had several limitations. Pooling INBs produced moderate to high heterogeneity particularly in UMICs. A meta-regression could be performed in a few pooling because of small number of studies particularly in UMIC, only a few factors could identify leading to subgroup analysis. Although we considered data from variable scenarios, we were still left with some estimated INBs that had no variances, and we had to ‘borrow’ the variances from similar studies. Although we limited the extent of heterogeneity by using several simulation methods, this was not possible for studies from UMICs. This highlights a need for uniformity of data reporting in economic analyses, particularly measures of dispersion, to enable SR-MA of economic evaluations. Our findings for rivaroxaban and edoxaban may be limited given the small number of evaluations published. Furthermore, the analyses from UMICs may also be affected by the quality of VKA monitoring; there is evidence that time in therapeutic range is lower in developing countries^130–133^ leading to higher rates of stroke and major bleeding with VKAs.^131 134^ Since clinical trial data under controlled conditions were used in the modelling, DOACs might potentially offer lower benefit in real-world practice for UMICs. The costs relative to hospitalisation are also much lower while drug prices tend to be more expensive in UMICs than HICs which may affect the cost-effectiveness balance of DOACs in UMICs. Changes in DOACs pricing such as the introduction of generic products may also influence our findings. In summary, our findings suggested that DOACs may be cost-effective relative to VKAs in HICs with TPP perspective given that DOACs are clinically non-inferior to VKAs. Our findings are based on studies with low risk of bias for most items, high risk in minor items should be less influenced and cancelled out in INB calculation. Further clinical and cost-effectiveness studies based on real-world clinical data from UMICs are clearly needed.

## Data Availability

No data are available. The authors confirm that the data supporting the findings of this study are available within the article and its supplementary materials.
