# Supplementary material for: Economic evaluation of direct oral anticoagulants (DOACs) versus vitamin K antagonists (VKAs) for stroke prevention in patients with atrial fibrillation: a systematic review and meta-analysis
Source: BMJ Evid Based Med. 2021 Oct 11;27(4):215–23. doi: 10.1136/bmjebm-2020-111634 (PMC9340051; doi:10.1136/bmjebm-2020-111634)
Supplement: Supplementary data [file bmjebm-2020-111634supp001.pdf]

Supplementary appendices

Economic evaluation of Direct Oral Anticoagulants (DOACs) versus Vitamin K Antagonists (VKAs) for stroke prevention in atrial fibrillation patients: a systematic review and meta-analysis

Noviyani R, Youngkong S, Nathisuwan S, Bagepally BS, Chaikledkaew U, Chaiyakunapruk N, McKay G, Attia J, Thakkinstian A

(All figures and tables in the supplementary appendices are created by the authors)

Online Supplementary Content

Contents

Appendix 1 Search strategies 1

Appendix 2 Data hamonisation and synthesis 3

Appendix 3 Characteristics of included studies and risk of bias assessment 6

eTable 3.1 List of excluded studies 6

eTable 3.2 Characteristics of the included studies 7

eTable 3.3 Risk of bias summary using the ECOBIAS checklist for each included study 8

Appendix 4 Results of meta-analyses: Dabigatran and Vitamin K Antagonists (VKAs) 12

eFigure 4.1 Pooling INBs comparing Dabigatran with VKAs in HICs estimated by Markov model, lifetime horizon and TPP. 12

eFigure 4.2 Pooling INBs comparing Dabigatran with VKAs in HICs estimated by Markov model, lifetime horizon and SP. 13

eFigure 4.3 Pooling INBs comparing Dabigatran with VKAs in UMICs estimated by Markov model, lifetime horizon and TPP. 14

eFigure 4.4 Pooling INBs comparing Dabigatran with VKAs in UMICs estimated by Markov model, lifetime horizon and SP. 15

eTable 4.1 Exploring sources of heterogeneity by a meta-regression analysis. 16

eFigure 4.5 Sub-group analysis by threshold of INB comparing Dabigatran with VKAs that estimated by Markov models with lifetime horizon and TPP in HICs. 18

eFigure 4.6 Sub-group analysis by grant source of INB comparing Dabigatran with VKAs that estimated by Markov models with lifetime horizon and TPP in HICs. 19

eFigure 4.7 Funnel plot comparing Dabigatran with VKAs that estimated by Markov models with lifetime horizon and TPP in HICs. 20

Appendix 5 Results of meta-analyses: Apixaban and Vitamin K Antagonists (VKAs) 21

eFigure 5.1 Pooling INBs comparing Apixaban with VKAs in HICs estimated by Markov model, lifetime horizon and TPP. 21

eFigure 5.2 Pooling INBs comparing Apixaban with VKAs in HICs estimated by Markov model, lifetime horizon and SP. 22

eFigure 5.3 Pooling INBs comparing Apixaban with VKAs in UMICs estimated by Markov model, lifetime horizon and TPP. 23

Noviyani R, *et al. BMJ EBM* 2022; 27:215–223. doi: 10.1136/bmjebm-2020-111634

eFigure 5.4 Pooling INBs comparing Apixaban with VKAs in UMICs estimated by Markov model, lifetime horizon and SP. 24

eTable 5.1 Exploring source of heterogeneity using a meta-regression analysis. 25

eFigure 5.5 Sub-group analysis by discount cost of INB comparing Apixaban with VKAs that estimated by Markov models with lifetime horizon and TPP in UMICs. 26

eFigure 5.6 Sub-group analysis by discount utility of INB comparing Apixaban with VKAs that estimated by Markov models with lifetime horizon and TPP in UMICs. 27

eFigure 5.7 Sub-group analysis by clinical data source of INB comparing Apixaban with VKAs that estimated by Markov models with lifetime horizon and TPP in UMICs. 28

eFigure 5.8 Sub-group analysis by utility data source of INB comparing Apixaban with VKAs that estimated by Markov models with lifetime horizon and TPP in UMICs. 29

eFigure 5.9 Funnel plot comparing Apixaban with VKAs that estimated by Markov models with lifetime horizon and TPP in HICs. 30

Appendix 6 Results of meta-analyses: Rivaroxaban and Vitamin K Antagonists (VKAs) 31

eFigure 6.1 Pooling INBs comparing Rivaroxaban with VKAs in HICs estimated by Markov model, lifetime horizon, and TPP. 31

eFigure 6.2 Pooling INBs comparing Rivaroxaban with VKAs in HICs estimated by Markov model, lifetime horizon, and SP. 32

eFigure 6.3 Pooling INBs comparing Rivaroxaban with VKAs in UMICs estimated by Markov model, lifetime horizon, and TPP. 33

eFigure 6.4 Pooling INBs comparing Rivaroxaban with VKAs in UMICs estimated by Markov model, lifetime horizon, and SP. 34

eTable 6.1 Exploring source of heterogeneity by a meta-regression analysis. 35

eFigure 6.5 Funnel plot comparing Rivaroxaban with VKAs that estimated by Markov models with lifetime horizon and TPP in HICs. 36

Appendix 7 Results of meta-analyses: Edoxaban and Vitamin K Antagonists (VKAs) 37

eFigure 7.1 Pooling INBs comparing Edoxaban with VKAs in HICs estimated by Markov model, lifetime horizon, and TPP. 37

eFigure 7.2 Pooling INBs comparing Edoxaban with VKAs in UMICs estimated by Markov model, lifetime horizon, and TPP. 38

eFigure 7.3 Pooling INBs comparing Edoxaban with VKAs in UMICs estimated by Markov model, lifetime horizon, and SP. 39

References 40

Noviyani R, *et al. BMJ EBM* 2022; 27:215–223. doi: 10.1136/bmjebm-2020-111634

Appendix 1 Search strategies

The search terms were constructed based on domains of population, intervention, comparator, and outcome (PICO) as below. Then these search terms were combined using Boolean operator OR within the same domains, and "AND" Boolean operator between domains of PICO as described.

Domain	Search terms
P	Atrial Fibrillation
I	NOAC<br>Oral Anticoagulants<br>Non Vitamin K Antagonists<br>Apixaban<br>Rivaroxaban<br>Dabigatran<br>Edoxaban
C	Warfarin<br>Vitamin K Antagonists<br>Acenocoumarol<br>Phenprocoumon<br>Coumarin
O	Incremental Net Benefit<br>Costs<br>Quality Adjusted Life Years<br>Incremental Cost Effectiveness Ratios
S	Economic evaluation

A) Search strategy from PubMed/Medline

DOMAIN	N of search PubMed	Search Terms
P	#1	Search **"atrial fibrillation"**
	#2	Search **"Atrial Fibrillation"[Mesh]**
	#3	#1 or #2
I	#4	Search **"noac*"**
	#5	Search **"oral anticoagulant*"**
	#6	Search **"non vitamin K antagonist*"**
	#7	Search **apixaban**
	#8	Search **rivaroxaban**
	#9	Search **dabigatran**
	#10	Search **edoxaban**
	#11	#4 or #5 or #6 or #7 or #8 or #9 or #10
O	#12	Search **"incremental net benefit"**
	#13	Search **"cost*"**
	#14	Search **"quality adjusted life year*"**
	#15	Search **"incremental cost effectiveness ratio*"**
	#16	Search **"economic evaluation"**
	#17	#12 or #13 or #14 or #15 or #16
PIO	#18	#3 and #11 and #18

B) Search strategy from Scopus

DOMAIN	N of search SCOPUS	Search Terms
P	#1	TITLE-ABS-KEY ("atrial fibrillation")
	#2	#1
I	#3	TITLE-ABS-KEY ("noac*")
	#4	TITLE-ABS-KEY ("oral anticoagulant*")
	#5	TITLE-ABS-KEY ("non vitamin k antagonist*")
	#6	TITLE-ABS-KEY (apixaban)
	#7	TITLE-ABS-KEY (rivaroxaban)
	#8	TITLE-ABS-KEY (dabigatran)
	#9	TITLE-ABS-KEY (edoxaban)
	#10	#3 or #4 or #5 or #6 or #7 or #8 or #9
O	#11	TITLE-ABS-KEY ("incremental net benefit")
	#12	TITLE-ABS-KEY ("cost*")
	#13	TITLE-ABS-KEY ("quality adjusted life year*")
	#14	TITLE-ABS-KEY ("incremental cost effectiveness ratio*")
	#15	TITLE-ABS-KEY ("economic evaluation")

Noviyani R, *et al. BMJ EBM* 2022; 27:215–223. doi: 10.1136/bmjebm-2020-111634

DOMAIN	N of search SCOPUS	Search Terms
	#16	#11 or #12 or #13 or #14 or #15
PIO	#17	#2 and #10 and #16

C) Search strategy from CEVR registry database

DOMAIN	N of search CEVR registry	Search Terms
Method	#1	Cost Effectiveness
	#2	Cost Utility
	#3	Economic Evaluation

Noviyani R, *et al. BMJ EBM* 2022; 27:215–223. doi: 10.1136/bmjebm-2020-111634

Appendix 2 Data hamonisation and synthesis

There were 4 steps of data hamonisation for further synthesis, namely currency conversion, data preparation, calculating INB and the variance of INB and statistical analysis by INB pooling.

A) Currency conversion

The relevant cost-effectiveness study reports economic terms in the currency units of each country at a certain time unit, so that currency conversion is needed for the standardization of monetary data. For the purposes of this analysis, all monetary units were converted to a single-year standard currency adjusted with purchasing power parity (PPP) 2019 to get PPP-adjusted US Dollars to the year of 2019. All monetary units except for country specific based threshold were adjusted to consumer price index and PPP conversion rates to 2019, using the formula:

$$Y_{\text{ppp2019}} = Y_{\text{€baseyear}} \times \left(\frac{\text{cpi}_{\text{€2019}}}{\text{cpi}_{\text{€base year}}} \times \frac{1}{\text{ppp2019}}\right)$$

Converting the value of the variance of monetary units using the formula:

$$Y_{\text{ppp2019}} = Y_{\text{€baseyear}} \times \left(\frac{\text{cpi}_{\text{€2019}}}{\text{cpi}_{\text{€base year}}} \times \frac{1}{\text{ppp2019}}\right)^2$$

B) Data Preparation

The next step is to complete the data needed to calculate the INB and its variance. In the formula for calculating the INB proposed by Crespo[1], the mean and the dispersion (up to 95% CI) of the costs and QALY are required. The data is obtained through data extraction, but many reports from cost-effectiveness studies in different forms that cause the data are not available, so to complete the lack of data, made scenarios.

There are five scenarios created based on the completeness of the data that cannot be extracted from included cost-effectiveness studies, namely:

- **Scenario 1**

Studies reported means along with measures of dispersion for costs, outcomes, ΔC, ΔE and Incremental Cost-Effectiveness Ratio (ICER). In this ideal situation, all the data required to calculate INB and its variance are available. Thus, the INB can be estimated as accordingly to the equation:[1]

INB= ΔE x (K - ICER) or INB= (K x ΔE) – ΔC

$$\text{Var (INB)} = K^2\sigma^2_{\Delta E} + \sigma^2_{ICER}$$

Where K is threshold, $\sigma^2_{\Delta E}$ is variance of ΔE and σ^2_{ICER} variance of ICER

- **Scenario 2**

Studies reported ICER along with 95%CI, the variance of ICER is calculated by formula:

$$UL_{ICER} = \mu + 1.96\ SE_{ICER}$$

$$SE_{ICER} = \frac{(UL_{ICER} - \mu\ )}{1.96}$$

Where UL is Upper Limit and μ is mean. Then, INB was calculated using above formula.

- **Scenario 3**

Studies reported mean as along with measures of dispersion (95% CI, SD or SE) of costs, outcomes, or, ΔC/ΔE but have not provided the ICER and its variance. Monte Carlo with 1000 simulation[2] would be used to simulate ΔC and ΔE data. Gamma distribution is used for ΔC and normal distribution is used for ΔE. If 95% CI is given, then the variance of ΔC and ΔE would be calculated but the covariance ($\rho_{\Delta C \Delta E}$) between, ΔC and ΔE are required to estimate using the simulated data. To calculate the variance of INB using the formula:

Var (INB) = $K^2\sigma^2_{\Delta E} + \sigma^2_{\Delta C} - 2K\rho_{\Delta C \Delta E}$

- **Scenario 4**

The studies have not reported any measures of dispersion but provided the Cost-Effective (CE) plane graphs for both intervention and comparator of interest as for a result of probabilistic sensitivity analysis (PSA).
The CE-plane graph is scatter plot of ΔC on Y-axis and ΔE on X-axis. data of ΔC and ΔE could be then extracted from the CE plane graph using Web-Plot Digitizer software version 4.2.[3]
As a result, mean of these ΔC and ΔE along with their variances and co-variances between ΔC and ΔE will be estimated leading to estimate the INB and its variance using the equation above.

- **Scenario 5**

Studies reported means of costs, outcomes, ΔC, ΔE or ICER but have not report neither the mean of dispersions nor the CE plane graph. The measure of dispersion would be taken from other studies that had reported data with following criteria:

1. Their ICERs were not much different, example: ±70% to ±85%
2. The studies were similar in intervention, comparator, time period, counties, perspective
3. The studies were in the same level of country's income, similar model inputs (eg, discount rate, time horizon, etc.)
4. If there are more than one study met the criteria, average of variances of those studies would be used.

C) Calculate INB and the variance of INB

INB is an outcome calculated using the formula developed by Crespo[1] namely INB = (K x Δ E) -ΔC where K is the threshold or willingness to pay, ΔE is the incremental QALY and ΔC is the incremental cost. A positive INB value indicates favoring intervention and a negative INB value indicates favoring comparator. The variance of INB is calculated using the formula as mentioned above.

D) Statistical analysis

Furthermore, pooling is carried out from INB and stratified based on country level of income. A total INB was estimated by using the random effect model by the Der Simonian and Laird [4] method if there is heterogeneity with the formula:

a. Random-effect model:

$$INB_p = \frac{\sum_{1-1}^{S} w_1^* - INB_1}{\sum_{1-1}^{S} w_1^*}$$

$$w_1^* = \frac{1}{[K^2\sigma^2_{\Delta E} + \sigma^2_{\Delta C} + 2K\rho_{\Delta E \Delta C}] + \tau^2}$$

$$\tau^2 = \frac{Q - (S - 1)}{\sum W_1 - \frac{\sum w_1^2}{\sum w_1}}$$

Q=0 if Q<S-1 (Q and s is number of comparisons)

and using inverse variance method if there is N heterogeneity with the formula:

b. Fixed-effect model:

$$INB_p = \frac{\sum_{1-1}^{S} w_1 - INB_1}{\sum_{1-1}^{S} w_1}$$

$$w_1 = \frac{1}{Var(INB)}$$

Noviyani R, *et al. BMJ EBM* 2022; 27:215–223. doi: 10.1136/bmjebm-2020-111634

$$w_1 = \frac{1}{K^2\sigma^2_{\Delta E} + \sigma^2_{\Delta C} + 2K\rho_{\Delta E \Delta C}}$$

The heterogeneity was assessed using the Cochrane-Q test and I^2 statistics. There was a heterogeneity if the I^2 statistics was greater than 25% or if the Cochrane-Q test p-value was <0.1. Here is the formula for Cochrane Q test and I^2:

c. Cochrane Q test

$$\text{Cochrane Q} = \sum_{i-1}^{s} w_1(\text{INB}_1 - \text{INBp})^2$$

Where W_1 is the inverse variance of INB_1, INB_1 is the individual studies, and INB_p is the pooled INB.

d. The I^2 statistic test

$$I^2 = 100\% \text{ x } \frac{Q - df}{Q}$$

Exploration of heterogeneity sources by considering some covariables such as thresholds, time horizons, and perspectives in a meta regression model for each covariable. A sensitivity analysis or subgroup analysis was applied according to these variables.

Publication was assessed using Egger's test and funnel plot. Publication can be determined if the funnel plot shows asymmetry or the p-value from Egger's test is less than 0.05. If there is asymmetry, the source of asymmetry will be explored using a contour-enhanced funnel plot. If missing studies in statistical non-significant areas means that there is a publication bias and if missing studies in both statistical non-significance and significant areas, means that caused by other reasons. All analyzes were performed using STATA version 16. Two-sided p <0.05 was considered statistically significant except for heterogeneity tests, in which p <0.10 was used.

Noviyani R, *et al. BMJ EBM* 2022; 27:215–223. doi: 10.1136/bmjebm-2020-111634

Appendix 3 Characteristics of included studies and risk of bias assessment

eTable 3.1 List of excluded studies

Full-text articles excluded, with reasons (N=14 studies):
Not DOACs nor VKAs (N=5)
Not interested outcomes (N=2)
Narrative reviews (N=3)
Conference abstracts (N=2)
Duplicated article (N=1)
Cannot retrieve full-text article (N=1)

Study	Reasons for exclusion
Abdullaev SP, 2019 [5]	Not DOACs nor VKAs
Belousov YB, 2012 [6]	Duplicated article
Bonet Pla A, 2013 [7]	Not interested outcomes
Kansal, 2013 [8]	Narrative reviews
Koretsune Y, 2018 [9]	Cannot retrieve full-text article
Monreal, 2017 [10]	Conference abstract
Nedogoda, 2017 [11]	Not interested outcomes
Rudakova AV, 2014 [12]	Conference abstract
Sorensen, 2013 [13]	Narrative reviews
Uetsuka Y, 2011 [14]	Narrative reviews
Vestergaard, 2015 [15]	Not DOACs nor VKAs
You JHS, 2012 [16]	Not DOACs nor VKAs
You JH, 2015 [17]	Not DOACs nor VKAs
You JHS, 2014 [18]	Not DOACs nor VKAs

eTable 3.2 Characteristics of the included studies (Created by the authors)

Category	Dabigatran versus VKAs						Rivaroxaban versus VKAs						Apixaban versus VKAs						Edoxaban versus VKAs					
	HIC		UMIC		LMIC		HIC		UMIC		LMIC		HIC		UMIC		LMIC		HIC		UMIC		LMIC	
	N	n	N	n	N	n	N	n	N	n	N	n	N	n	N	n	N	n	N	n	N	n	N	n
Perspective																								
Third-party payer	32	38	8	9	0	0	20	22	6	6	1	1	26	28	7	7	0	0	11	13	2	2	0	0
Societal	6	6	4	4	0	0	4	4	4	4	0	0	3	3	4	4	0	0	1	1	3	3	0	0
Patient	4	4	0	0	0	0	2	4	0	0	0	0	2	2	0	0	0	0	1	1	0	0	0	0
Model type																								
Markov	37	45	9	13	0	0	24	26	7	10	1	1	28	30	8	11	0	0	13	15	3	5	0	0
Discrete event simulation	3	3	0	0	0	0	2	2	0	0	0	0	2	2	0	0	0	0	0	0	0	0	0	0
EE alongside clinical trial	0	0	0	0	0	0	0	0	0	0	0	0	1	1	0	0	0	0	0	0	0	0	0	0
Time horizon																								
Lifetime	39	47	8	11	0	0	26	28	7	10	1	1	31	33	7	10	0	0	13	15	3	5	0	0
Not lifetime	1	1	1	2	0	0	0	0	0	0	0	0	0	0	1	1	0	0	0	0	0	0	0	0
Discount rate for cost																								
Not reported	3	4	0	0	0	0	2	3	0	0	0	0	2	3	0	0	0	0	1	2	0	0	0	0
≤3%	22	26	5	8	0	0	15	15	6	9	1	1	16	16	6	9	0	0	9	9	3	5	0	0
>3%	15	18	4	5	0	0	9	10	1	1	0	0	13	14	2	2	0	0	3	4	0	0	0	0
Discount rate for utility																								
Not reported	2	3	1	1	0	0	1	2	1	1	0	0	1	2	0	0	0	0	1	2	0	0	0	0
≤3%	23	27	5	8	0	0	16	16	6	9	1	1	17	17	6	9	0	0	9	9	3	5	0	0
>3%	15	18	3	4	0	0	9	10	0	0	0	0	13	14	2	2	0	0	3	4	0	0	0	0
Clinical data source																								
Published literature	35	42	6	8	0	0	20	21	6	8	1	1	23	24	7	9	0	0	10	11	3	5	0	0
Published literature-evidence synthesis	2	3	1	2	0	0	2	3	1	2	0	0	2	3	1	2	0	0	1	2	0	0	0	0
Published literature-registry database	2	2	1	2	0	0	2	2	0	0	0	0	4	4	0	0	0	0	1	1	0	0	0	0
Evidence synthesis	1	1	0	0	0	0	1	1	0	0	0	0	1	1	0	0	0	0	1	1	0	0	0	0
Registry database	0	0	1	1	0	0	1	1	0	0	0	0	1	1	0	0	0	0	0	0	0	0	0	0
Utility data source																								
Published literature	38	46	8	11	0	0	24	26	6	8	1	1	30	32	7	9	0	0	12	14	2	3	0	0
Published literature-registry database	0	0	1	2	0	0	1	1	1	2	0	0	1	1	1	2	0	0	1	1	1	2	0	0
Survey	2	2	0	0	0	0	1	1	0	0	0	0	0	0	0	0	0	0	0	0	0	0	0	0
Currency year																								
2008-2013	33	41	3	5	0	0	17	19	2	3	1	1	20	22	3	4	0	0	5	7	0	0	0	0
2014-2019	7	7	6	8	0	0	9	9	5	7	0	0	11	11	5	7	0	0	8	8	3	5	0	0
Cost-effectiveness threshold																								
Country specific	35	43	2	3	0	0	22	24	2	3	0	0	26	28	2	3	0	0	9	11	2	3	0	0
GDP based	4	4	7	10	0	0	3	3	5	7	1	1	3	3	6	8	0	0	2	2	1	2	0	0
Others	1	1	0	0	0	0	1	1	0	0	0	0	2	2	0	0	0	0	2	2	0	0	0	0
Cost-effectiveness result																								
Cost-effective	30	38	3	4	0	0	17	19	1	1	0	0	29	31	3	3	0	0	11	13	0	0	0	0
Not cost-effective	10	10	6	9	0	0	9	9	6	9	1	1	2	2	5	8	0	0	2	2	3	5	0	0

Abbreviations: VKAs, Vitamin K-Antagonists; EE, Economic Evaluation; GDP, Gross Domestic Product; HIC, High Income Country; UMIC, Upper-Middle Income Country; LMIC, Lower-Middle Income Country; N, number of studies; n, number of comparisons.

eTable 3.3 Risk of bias summary using the ECOBIAS checklist for each included study (Created by the authors)

Author	Part A Overall checklist for bias in economic evaluation											Part B Model-specific aspects of bias in economic evaluation										
												I[1]			II[2]							III[3]
	Narrow perspective bias	Inefficient comparator bias*	Cost measurement omission bias	Intermittent data collection bias	Invalid valuation bias	Ordinal ICER bias	Double-counting bias	Inappropriate discounting bias	Limited sensitivity analysis bias§	Sponsor bias	Reporting and dissemination bias	Structural assumptions bias	N treatment comparator bias*	Wrong model bias	Limited time horizon bias	Bias related to data identification	Bias related to baseline data	Bias related to treatment effects	Bias related to quality-of-life weights (utilities)	Non-transparent data incorporation bias	Limited to scope bias	Bias related to internal consistency
	1	2	3	4	5	6	7	8	9	10	11	12	13	14	15	16	17	18	19	20	21	22
Pink J, 2011[19]	N	Y	Y	Y	Y	Y	N	Y	Y	Y	N	Y	Y	Y	Y	Y	Y	Y	Y	Y	Y	N
Dilokthornsakul, P, 2019[20]	Y	Y	Y	Y	Y	Y	N	Y	Y	Y	N	Y	Y	Y	Y	Y	Y	Y	Y	Y	Y	N
Harrington A, 2013[21]	N	Y	Y	Y	Y	Y	N	N	Y	Y	N	Y	Y	Y	Y	Y	Y	Y	Y	Y	Y	N
Lopez, 2017[22]	N	Y	Y	Y	Y	Y	N	NA	Y	Y	N	Y	Y	Y	Y	Y	Y	Y	Y	Y	Y	N
Verhoef TI, 2014[23]	Y	Y	Y	Y	Y	Y	N	Y	Y	Y	N	Y	Y	Y	Y	Y	Y	Y	Y	Y	Y	N
StevaNvic J, 2014[24]	N	Y	Y	Y	Y	Y	N	Y	Y	Y	N	Y	Y	Y	Y	Y	Y	Y	Y	Y	Y	N
Ademi Z, 2015[25]	N	Y	P	Y	P	Y	N	N	Y	Y	N	Y	Y	Y	Y	Y	Y	Y	Y	P	Y	N
Andrikopoulos GK, 2013[26]	N	Y	Y	Y	Y	Y	N	N	Y	Y	N	Y	Y	Y	Y	Y	Y	Y	Y	Y	Y	N
Shah A, 2016[27]	N	Y	Y	Y	Y	Y	N	N	Y	Y	N	Y	Y	Y	Y	P	Y	Y	Y	Y	Y	N
Kamae I, 2015[28]	Y	Y	Y	Y	Y	Y	N	Y	Y	Y	N	Y	Y	Y	Y	P	Y	Y	Y	Y	Y	N
Lip GYH, 2014[29]	Y	Y	Y	Y	Y	Y	N	N	Y	Y	N	Y	Y	Y	Y	Y	Y	Y	Y	Y	Y	N
Jarungsuccess S, 2014[30]	Y	Y	Y	Y	Y	Y	N	Y	Y	Y	N	Y	Y	Y	Y	Y	Y	Y	Y	Y	Y	N
Athanasakis K, 2015[31]	Y	Y	Y	Y	Y	Y	N	N	Y	Y	N	Y	Y	Y	Y	Y	Y	Y	Y	Y	Y	N
Coyle D, 2013[32]	N	Y	Y	Y	Y	Y	N	N	Y	Y	N	Y	Y	Y	Y	Y	Y	Y	Y	Y	Y	N
Pletscher M, 2013[33]	Y	Y	Y	Y	Y	Y	N	N	Y	Y	N	Y	Y	Y	Y	P	P	Y	Y	P	Y	N
Miller JD, 2014[34]	Y	Y	Y	Y	Y	Y	N	Y	Y	Y	N	Y	Y	Y	Y	Y	Y	Y	Y	Y	Y	N
Magnuson, EA, 2015[35]	N	Y	Y	Y	Y	Y	N	Y	Y	Y	N	Y	Y	Y	Y	Y	Y	Y	Y	Y	Y	N
Athanasakis K, 2017[36]	Y	Y	Y	Y	Y	Y	N	N	Y	Y	N	Y	Y	Y	Y	Y	Y	Y	Y	Y	Y	N
Kamel H, 2012[37]	Y	Y	Y	Y	Y	Y	N	NA	Y	N	N	Y	Y	Y	Y	Y	Y	Y	Y	Y	Y	Y
Pink J, 2014[38]	N	Y	Y	Y	Y	Y	N	N	Y	Y	N	Y	Y	Y	Y	Y	Y	Y	Y	Y	Y	N
Dorian P, 2014[39]	Y	Y	Y	Y	Y	Y	N	N	Y	Y	N	Y	Y	Y	Y	Y	Y	Y	Y	Y	Y	N
Lanitis T, 2014[40]	Y	Y	Y	Y	Y	Y	N	Y	Y	Y	N	Y	Y	Y	Y	Y	Y	Y	Y	Y	Y	N
Canestaro WJ, 2013[41]	Y	P	Y	Y	Y	Y	N	N	Y	Y	P	Y	Y	Y	Y	Y	Y	Y	Y	Y	Y	N
Wisloff , 2014[42]	N	Y	Y	Y	Y	Y	N	Y	Y	Y	N	Y	Y	Y	Y	Y	Y	Y	Y	Y	Y	N
Janzic A, 2014[43]	N	Y	Y	Y	P	Y	N	N	Y	Y	N	Y	Y	Y	Y	P	Y	Y	Y	P	Y	N
Zheng Y, 2014[44]	N	Y	Y	Y	Y	Y	Y	Y	Y	Y	N	Y	Y	Y	Y	N	Y	Y	Y	Y	Y	Y
Baron Esquivias G, 2013[45]	Y	Y	Y	Y	Y	Y	N	N	Y	Y	N	Y	Y	Y	Y	Y	Y	Y	Y	Y	Y	N
Giorgi MA, 2015[46]	N	Y	Y	Y	Y	Y	N	Y	Y	Y	N	Y	Y	Y	Y	Y	Y	Y	Y	Y	Y	N
Pradelli L, 2014[47]	N	Y	Y	Y	Y	Y	N	N	Y	Y	N	Y	Y	Y	Y	Y	Y	P	Y	P	Y	N
Li X, 2015[48]	N	Y	Y	Y	Y	Y	N	Y	Y	Y	N	Y	Y	Y	Y	Y	Y	Y	Y	Y	Y	N
Lanitis T, 2014[49]	Y	Y	Y	Y	Y	Y	N	Y	Y	Y	N	Y	Y	Y	Y	Y	Y	Y	Y	Y	Y	N
Krejczy M,	N	Y	Y	Y	Y	Y	N	N	Y	N	N	Y	Y	Y	Y	Y	Y	Y	Y	Y	Y	N

Noviyani R, *et al. BMJ EBM* 2022; 27:215–223. doi: 10.1136/bmjebm-2020-111634

Author	Part A Overall checklist for bias in economic evaluation											Part B Model-specific aspects of bias in economic evaluation										
												I[1]			II[2]							III[3]
	Narrow perspective bias	Inefficient comparator bias*	Cost measurement omission bias	Intermittent data collection bias	Invalid valuation bias	Ordinal ICER bias	Double-counting bias	Inappropriate discounting bias	Limited sensitivity analysis bias§	Sponsor bias	Reporting and dissemination bias	Structural assumptions bias	N treatment comparator bias*	Wrong model bias	Limited time horizon bias	Bias related to data identification	Bias related to baseline data	Bias related to treatment effects	Bias related to quality-of-life weights (utilities)	Non-transparent data incorporation bias	Limited to scope bias	Bias related to internal consistency
	1	2	3	4	5	6	7	8	9	10	11	12	13	14	15	16	17	18	19	20	21	22
2014[50]																						
Lee S, 2012[51]	N	Y	Y	Y	Y	Y	N	N	Y	Y	N	Y	Y	Y	Y	Y	Y	Y	Y	Y	Y	N
Rognoni C, 2013[52]	Y	Y	Y	Y	Y	Y	N	N	Y	Y	N	Y	Y	Y	Y	Y	Y	Y	Y	Y	Y	N
Kongnakorn T, 2014[53]	Y	Y	Y	Y	Y	Y	N	Y	Y	Y	N	Y	Y	Y	Y	Y	Y	Y	Y	Y	Y	N
Mensch A, 2015[54]	Y	Y	Y	Y	Y	Y	N	N	Y	P	N	Y	Y	Y	Y	Y	Y	Y	Y	Y	Y	N
Nguyen E, 2016[55]	Y	Y	Y	Y	Y	Y	N	N	Y	P	N	Y	Y	Y	Y	Y	Y	Y	Y	Y	Y	N
Rattanachotpanit T, 2019[56]	Y	Y	Y	Y	Y	Y	N	N	Y	Y	N	Y	Y	Y	Y	Y	Y	Y	Y	Y	Y	N
Rognoni C, 2015[57]	N	Y	Y	Y	Y	Y	N	Y	Y	Y	N	Y	Y	Y	Y	P	Y	Y	Y	P	Y	N
Costa J, 2015[58]	Y	Y	Y	Y	Y	Y	N	Y	Y	Y	N	Y	Y	Y	Y	Y	Y	Y	Y	Y	Y	N
Kleintjens J, 2013[59]	Y	Y	Y	Y	Y	Y	N	Y	Y	Y	N	Y	Y	Y	Y	Y	Y	Y	Y	Y	Y	N
Sorensen SV, 2011[60]	N	Y	Y	Y	Y	Y	N	Y	Y	Y	N	Y	Y	Y	Y	Y	Y	Y	Y	Y	Y	N
Zhao YJ, 2016[61]	N	Y	Y	Y	Y	Y	N	N	Y	Y	N	Y	Y	Y	Y	Y	Y	Y	Y	Y	Y	N
Lekuona I, 2019[62]	N	Y	Y	Y	Y	Y	N	N	Y	Y	N	Y	Y	Y	Y	Y	Y	Y	Y	Y	Y	N
Lanas F, 2017[63]	N	Y	Y	Y	Y	Y	N	Y	Y	Y	P	Y	Y	Y	Y	Y	Y	Y	Y	Y	Y	N
Wu B, 2014[64]	Y	Y	Y	Y	Y	Y	N	N	Y	Y	N	Y	Y	Y	Y	Y	Y	Y	Y	Y	Y	N
Langkilde LK, 2012[65]	Y	Y	Y	Y	Y	Y	Y	Y	Y	Y	N	Y	Y	Y	Y	P	P	P	Y	P	Y	Y
Gonzalez-Juanatey JR, 2012[66]	N	Y	Y	Y	Y	Y	N	N	Y	N	N	Y	Y	Y	Y	P	P	P	P	P	Y	N
Chang CH, 2014[67]	N	Y	Y	Y	Y	Y	N	N	Y	Y	N	Y	Y	Y	Y	Y	Y	Y	Y	Y	Y	N
Kim H, 2019[68]	Y	Y	Y	Y	Y	Y	N	N	Y	Y	N	Y	Y	Y	Y	P	Y	Y	Y	P	Y	N
Vilain KA, 2017[69]	N	Y	Y	Y	Y	Y	N	Y	Y	Y	N	Y	Y	Y	Y	Y	Y	Y	Y	Y	Y	N
Carles M, 2015[70]	Y	Y	Y	Y	Y	Y	N	N	Y	Y	N	Y	Y	Y	Y	Y	Y	Y	Y	Y	Y	N
Wang Y, 2014[71]	Y	Y	Y	Y	Y	Y	N	N	Y	Y	N	Y	Y	Y	Y	Y	Y	Y	Y	Y	Y	N
Hospodar Ar, 2018[72]	N	Y	Y	Y	Y	Y	N	NA	Y	Y	N	Y	Y	Y	Y	Y	Y	Y	Y	Y	Y	N
Liu CY, 2017[73]	N	Y	Y	Y	P	Y	N	N	Y	Y	N	Y	Y	Y	Y	P	P	Y	Y	P	Y	N
Lip GYH, 2015[74]	N	Y	Y	Y	Y	Y	N	N	Y	Y	N	Y	Y	Y	Y	Y	Y	Y	Y	Y	Y	N
Hulst MV, 2017[75]	Y	Y	Y	Y	Y	Y	Y	Y	Y	Y	N	Y	Y	Y	Y	P	P	P	Y	P	Y	N
Mendoza JA, 2019[76]	N	Y	Y	Y	P	Y	N	N	Y	N	N	Y	Y	Y	P	Y	Y	Y	Y	P	Y	N
Lee S, 2012[77]	N	P	Y	Y	Y	Y	N	N	Y	N	N	Y	Y	Y	Y	Y	Y	Y	Y	Y	Y	N
Wouters H, 2013[78]	N	Y	Y	Y	Y	Y	N	Y	Y	Y	N	Y	Y	Y	Y	P	N	N	N	N	Y	N
Kourlaba G, 2014[79]	N	Y	Y	Y	Y	Y	N	Y	Y	Y	N	Y	Y	Y	Y	Y	Y	Y	Y	Y	Y	N
Kansal AR, 2012[80]	N	Y	Y	Y	Y	Y	N	N	Y	Y	P	Y	Y	Y	Y	Y	Y	Y	Y	Y	Y	N
Kamel H, 2012[81]	Y	Y	Y	Y	Y	Y	N	N	Y	N	P	Y	Y	Y	NA	P	Y	Y	Y	P	Y	N
Galvani G, 2015[82]	Y	Y	N	Y	N	Y	N	NA	N	N	N	Y	Y	Y	Y	N	N	N	N	N	N	N
Hallinen T,	N	Y	Y	Y	P	Y	N	N	Y	Y	N	Y	Y	Y	Y	P	Y	P	Y	P	Y	N

Noviyani R, *et al. BMJ EBM* 2022; 27:215–223. doi: 10.1136/bmjebm-2020-111634

Author	Part A Overall checklist for bias in economic evaluation											Part B Model-specific aspects of bias in economic evaluation										
												I[1]			II[2]							III[3]
	Narrow perspective bias	Inefficient comparator bias*	Cost measurement omission bias	Intermittent data collection bias	Invalid valuation bias	Ordinal ICER bias	Double-counting bias	Inappropriate discounting bias	Limited sensitivity analysis bias§	Sponsor bias	Reporting and dissemination bias	Structural assumptions bias	N treatment comparator bias*	Wrong model bias	Limited time horizon bias	Bias related to data identification	Bias related to baseline data	Bias related to treatment effects	Bias related to quality-of-life weights (utilities)	Non-transparent data incorporation bias	Limited to scope bias	Bias related to internal consistency
	1	2	3	4	5	6	7	8	9	10	11	12	13	14	15	16	17	18	19	20	21	22
2016[83]																						
Bergh M, 2013[84]	Y	Y	Y	Y	P	Y	N	NA	P	Y	N	Y	Y	Y	Y	N	N	N	N	N	N	N
Dwiprahasto I, 2019[85]	N	Y	Y	Y	P	Y	N	N	Y	Y	N	Y	Y	Y	N	P	P	P	Y	P	Y	N
Cowper PA, 2017[86]	N	Y	Y	Y	Y	Y	N	N	Y	Y	N	Y	Y	Y	Y	Y	Y	Y	Y	Y	Y	N
De Souza CPR, 2015[87]	N	Y	Y	Y	Y	Y	N	Y	Y	Y	N	Y	Y	Y	N	Y	Y	Y	Y	Y	Y	N
Salcedo J, 2019[88]	Y	Y	Y	Y	Y	Y	N	N	Y	Y	N	Y	Y	Y	Y	Y	Y	Y	Y	Y	Y	N
Salata BM, 2016[89]	Y	Y	Y	Y	Y	Y	N	N	Y	Y	N	Y	Y	Y	Y	Y	Y	Y	Y	Y	Y	N
Shah SV, 2011[90]	Y	Y	Y	Y	Y	Y	N	N	Y	Y	N	Y	Y	Y	N	Y	Y	Y	Y	Y	Y	N
Freeman JV, 2011[91]	Y	Y	Y	Y	Y	Y	N	N	Y	Y	N	Y	Y	Y	Y	P	Y	Y	Y	P	Y	N
Chevalier J, 2014[92]	N	Y	Y	Y	Y	Y	N	Y	Y	Y	N	Y	Y	Y	Y	Y	Y	Y	Y	Y	Y	N
Nshimyumukiza L, 2013[93]	N	Y	Y	Y	Y	Y	N	N	Y	Y	N	Y	Y	Y	Y	Y	Y	Y	Y	Y	Y	N
Hernandez I, 2017[94]	N	Y	Y	Y	Y	Y	N	N	Y	Y	N	Y	Y	Y	Y	Y	Y	Y	Y	Y	Y	N
Peng S, 2017[95]	N	Y	Y	Y	Y	Y	N	Y	Y	Y	N	Y	Y	Y	Y	Y	Y	Y	Y	Y	Y	N
Clemens A, 2014[96]	N	Y	Y	Y	Y	Y	N	N	P	Y	N	Y	Y	Y	Y	P	Y	Y	N	P	P	N
Kansal AR, 2012[97]	N	Y	Y	Y	Y	Y	N	Y	Y	Y	N	Y	Y	Y	Y	Y	Y	Y	Y	Y	Y	N
Davidson T, 2013[98]	Y	Y	Y	Y	Y	Y	N	N	Y	Y	N	Y	Y	Y	Y	Y	Y	Y	Y	Y	Y	N
Morais J, 2014[99]	Y	Y	Y	Y	P	Y	N	Y	Y	N	N	Y	Y	Y	Y	P	Y	Y	P	P	Y	N
Hersi AS, 2019[100]	Y	Y	Y	Y	Y	Y	Y	N	Y	Y	N	Y	Y	Y	Y	Y	Y	Y	Y	Y	Y	Y
Krejczy M, 2014[101]	N	Y	Y	Y	Y	Y	N	N	Y	P	N	Y	Y	Y	Y	Y	Y	Y	Y	Y	Y	Y
Pink J, 2013[102]	Y	Y	N	Y	N	Y	N	NA	Y	Y	N	Y	Y	Y	Y	P	Y	Y	Y	P	Y	N
Thom HHZ, 2019[103]	Y	Y	Y	Y	Y	Y	N	NA	Y	Y	N	Y	Y	Y	Y	Y	Y	Y	Y	Y	Y	N
You JHS, 2013[104]	N	Y	Y	Y	P	Y	N	N	Y	Y	N	Y	Y	Y	Y	Y	Y	Y	Y	P	Y	N
Yong Fa-C, 2016[105]	N	Y	Y	Y	P	Y	N	N	Y	N	N	Y	Y	Y	N	P	Y	Y	Y	P	Y	N
Fontcuberta CC, 2015[106]	Y	Y	Y	Y	Y	Y	N	N	Y	Y	N	Y	Y	Y	Y	Y	Y	Y	Y	Y	Y	N
Garcia-Pena AA, 2017[107]	Y	Y	Y	Y	P	Y	N	N	Y	Y	P	Y	Y	Y	Y	Y	Y	Y	Y	P	Y	N
Miguel LS, 2016[108]	Y	Y	Y	Y	P	Y	N	Y	Y	Y	N	Y	Y	Y	Y	Y	Y	Y	Y	P	Y	N
Miguel LS, 2013[109]	Y	Y	Y	Y	P	Y	N	Y	Y	Y	N	Y	Y	Y	Y	Y	Y	Y	Y	P	Y	N
Ravasio R, 2014[110]	N	Y	Y	Y	Y	Y	Y	N	Y	Y	N	Y	Y	Y	Y	Y	Y	Y	Y	Y	Y	Y
Triana JJ, 2016[111]	N	Y	N	Y	N	Y	N	N	Y	Y	N	Y	Y	Y	Y	N	N	N	N	N	Y	N
Rudakova AV, 2014[112]	N	Y	N	Y	N	Y	N	N	P	Y	N	Y	Y	Y	Y	N	N	N	N	N	P	N
Oyaguez I, 2019[113]	Y	Y	Y	Y	Y	Y	Y	Y	Y	Y	N	Y	Y	Y	Y	Y	Y	Y	Y	Y	Y	Y
Hori M, 2019[114]	Y	Y	Y	Y	Y	Y	N	Y	Y	Y	N	Y	Y	Y	Y	Y	Y	Y	Y	Y	Y	N
Ng SS, 2020[115]	Y	Y	Y	Y	Y	Y	Y	Y	Y	Y	N	Y	Y	Y	Y	Y	Y	Y	Y	Y	Y	Y

Author	Part A Overall checklist for bias in economic evaluation											Part B Model-specific aspects of bias in economic evaluation										
												I[1]			II[2]							III[3]
	Narrow perspective bias	Inefficient comparator bias*	Cost measurement omission bias	Intermittent data collection bias	Invalid valuation bias	Ordinal ICER bias	Double-counting bias	Inappropriate discounting bias	Limited sensitivity analysis bias§	Sponsor bias	Reporting and dissemination bias	Structural assumptions bias	N treatment comparator bias*	Wrong model bias	Limited time horizon bias	Bias related to data identification	Bias related to baseline data	Bias related to treatment effects	Bias related to quality-of-life weights (utilities)	Non-transparent data incorporation bias	Limited to scope bias	Bias related to internal consistency
	1	2	3	4	5	6	7	8	9	10	11	12	13	14	15	16	17	18	19	20	21	22
De Jong LA, 2019[116]	Y	Y	Y	Y	Y	Y	N	Y	Y	Y	N	Y	Y	Y	Y	Y	Y	Y	Y	Y	Y	N
de Pouvourville G, 2019[117]	Y	Y	Y	Y	Y	Y	Y	Y	Y	Y	N	Y	Y	Y	Y	Y	Y	Y	Y	Y	Y	N
Taborsky M, 2019[118]	N	Y	Y	Y	Y	Y	N	Y	Y	N	N	Y	Y	Y	Y	Y	Y	Y	Y	Y	Y	N

Abbreviations: N, No-high risk of bias; Y, Yes–low risk of bias; P, Partly bias; NA, Not Available.

[1] the bias related to structure

[2] the bias related to data

[3] the bias related to consistency

Appendix 4 Results of meta-analyses: Dabigatran and Vitamin K Antagonists (VKAs)

A) Pooling INBs

eFigure 4.1 Pooling INBs comparing Dabigatran with VKAs in HICs estimated by Markov model, lifetime horizon and TPP. (Created by the authors)

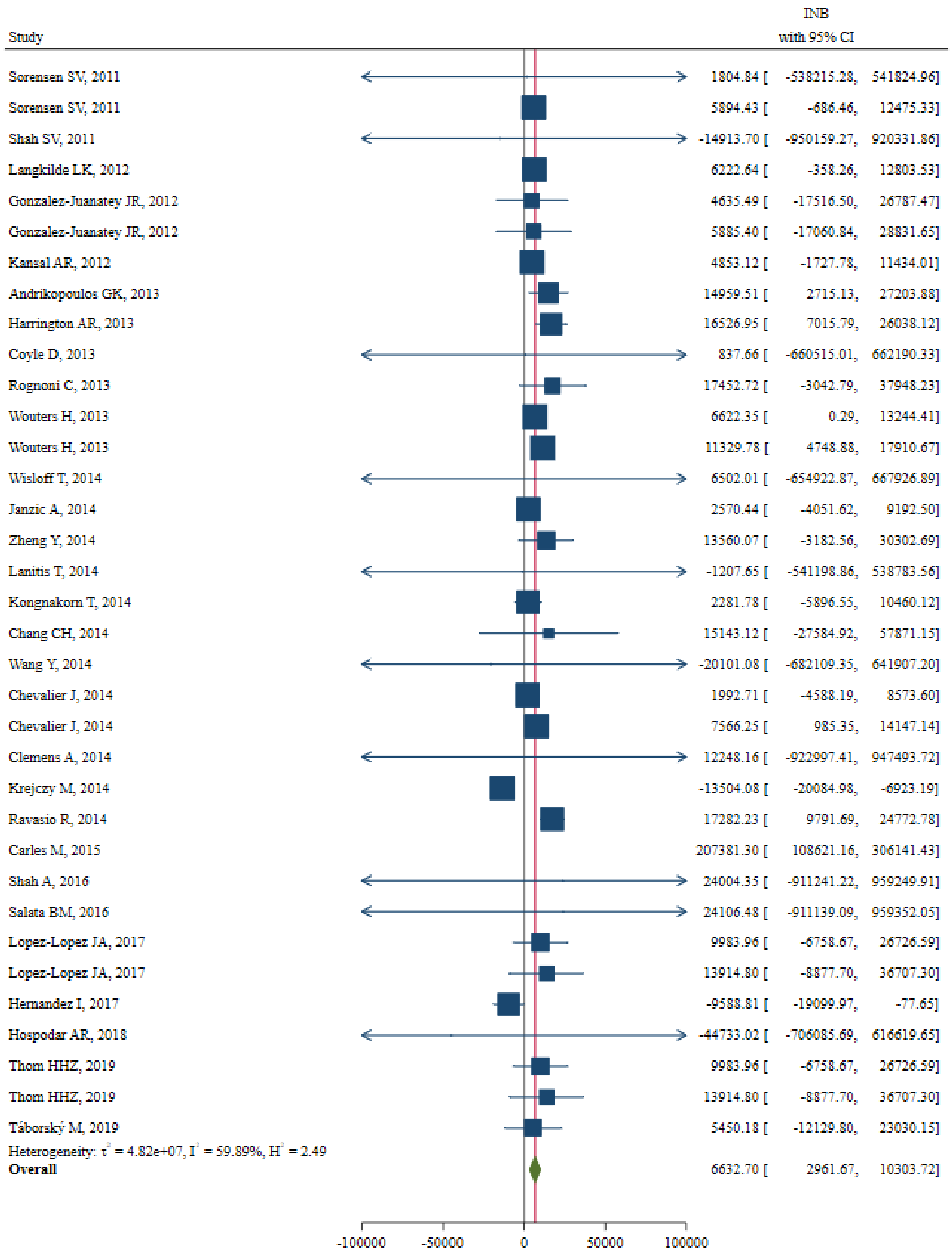

Abbreviations: INBs, Incremental Net Benefits; VKAs, Vitamin K Antagonists; HICs, High-Income Countries; TPP, Third-party payer perspective.

eFigure 4.2 Pooling INBs comparing Dabigatran with VKAs in HICs estimated by Markov model, lifetime horizon and SP. (Created by the authors)

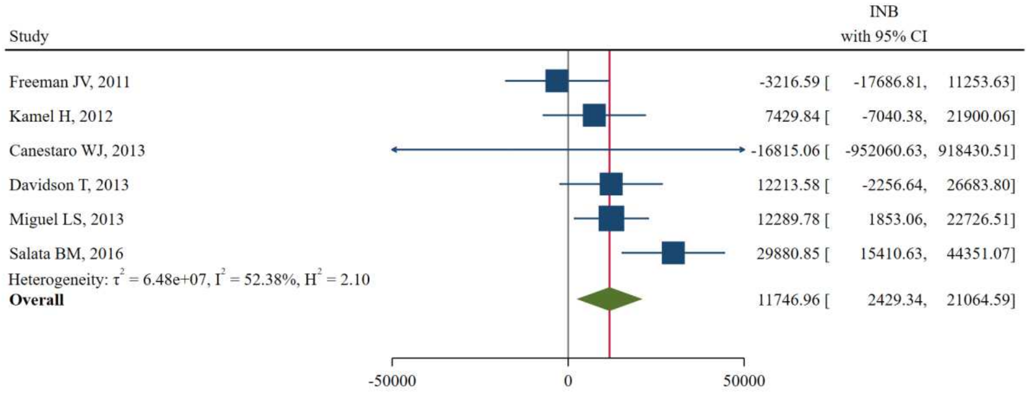

Abbreviations: INBs, Incremental Net Benefits; VKAs, Vitamin K Antagonists; HICs, High-Income Countries; SP, Societal perspective**.**

eFigure 4.3 Pooling INBs comparing Dabigatran with VKAs in UMICs estimated by Markov model, lifetime horizon and TPP. (Created by the authors)

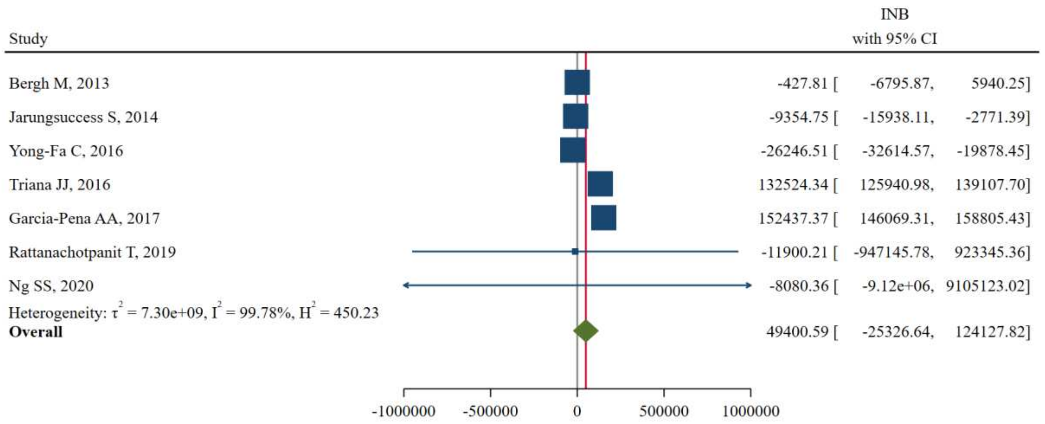

Abbreviations: INBs, Incremental Net Benefits; VKAs, Vitamin K Antagonists; UMICs, Upper Middle-Income Countries; TPP, Third-party payer perspective**.**

eFigure 4.4 Pooling INBs comparing Dabigatran with VKAs in UMICs estimated by Markov model, lifetime horizon and SP. (Created by the authors)

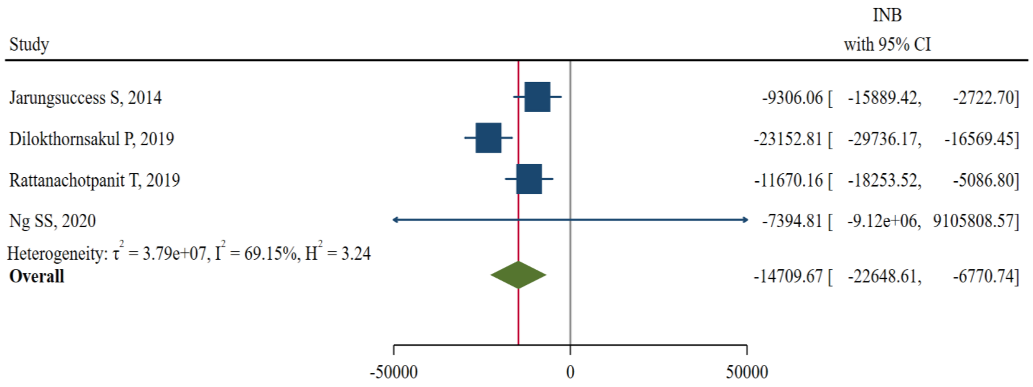

Abbreviations: INBs, Incremental Net Benefits; VKAs, Vitamin K Antagonists; UMICs, Upper Middle-Income Countries; SP, Societal perspective**.**

Noviyani R, *et al. BMJ EBM* 2022; 27:215–223. doi: 10.1136/bmjebm-2020-111634

B) Meta-regression analysis

eTable 4.1 Exploring sources of heterogeneity by a meta-regression analysis. (Created by the authors)

**Factors**	**Coefficient**	**SE**	**P-value**	**I^2 (%)**
Dabigatran vs VKAs in HICs Markov-TPP-LT				
Model without factors	6,632.695	1,873.005	0.001	59.89
WTP Threshold				
18,963.34-30,539.2 vs >50,000	303.4636	4,365.701	0.945	60.92
34,482.76-50,000 vs >50,000	-1,828.197	5,370.961	0.736	
Discount cost				
≥3% vs <3%	-1,541.342	4,002.778	0.703	61.02
Discount utility				
≥3% vs <3%	-1,541.342	4,002.778	0.703	61.02
Clinical data source				
PL Evidence Synthesis vs PL	5,208.721	8,893.681	0.562	61.81
PL Registry database vs PL	8,760.234	23,161.07	0.708	61.81
Funding source				
Pharma-grant vs no data	7,222.606	3,850.02	0.070	51.89
Non-pharma-grant vs no data	10,405.1	6,655.359	0.128	
Dabigatran vs VKAs in HICs Markov SP LT				
Model without factors	11,746.96	4,753.977	0.056	52.38
WTP Threshold				
50,704.23-100,000 vs < 50,704.23	14,627.96	8,058.964	0.144	30.72
Discount cost				
≥3% vs <3%	718.2315	13,759.11	0.961	61.86
Discount utility				
≥3% vs <3%	718.2315	13,759.11	0.961	61.86
Dabigatran vs VKAs in UMICs Markov TPP LT				
Model without factors	49,400.59	38,126.84	0.243	99.78

Factors	Coefficient	SE	P-value	I^2 (%)
WTP Threshold				
43,695.49-770,414.2 vs 12959.5-18498.4	91,224.38	73,396.51	0.269	99.72
Discount cost				
≥3% vs <3%	-34,678.48	86,468.17	0.705	99.80
Discount utility				
≥3% vs <3%	-3,606.83	104,057.4	0.974	99.80
Clinical data source				
PL-Evidence synthesis vs PL	-50,572.68	111,273.6	0.673	99.77
Registry database vs PL	91,306.41	111,273.6	0.458	99.77
Utility data source				
PL-Registry database vs PL	-61,682.35	486,276.7	0.904	99.81
Grant source				
Pharma-grant vs No data	27,106.04	89,094.74	0.776	99.85
Non-pharma-grant vs No data	-50,839.9	490,291.6	0.922	99.85

Abbreviations: HICs, High-Income Countries; LT, lifetime; PL, Published Literature; SE, Standard Error; SP, Societal Perspective; TPP, Third-party payer perspective; UMICs, Upper Middle-Income Countries; VKAs, Vitamin K Antagonists, VS, versus; WTP, Willingness-to-Pay.

Noviyani R, *et al. BMJ EBM* 2022; 27:215–223. doi: 10.1136/bmjebm-2020-111634

C) Sub-group Analysis

eFigure 4.5 Sub-group analysis by threshold of INB comparing Dabigatran with VKAs that estimated by Markov models with lifetime horizon and TPP in HICs. (Created by the authors)

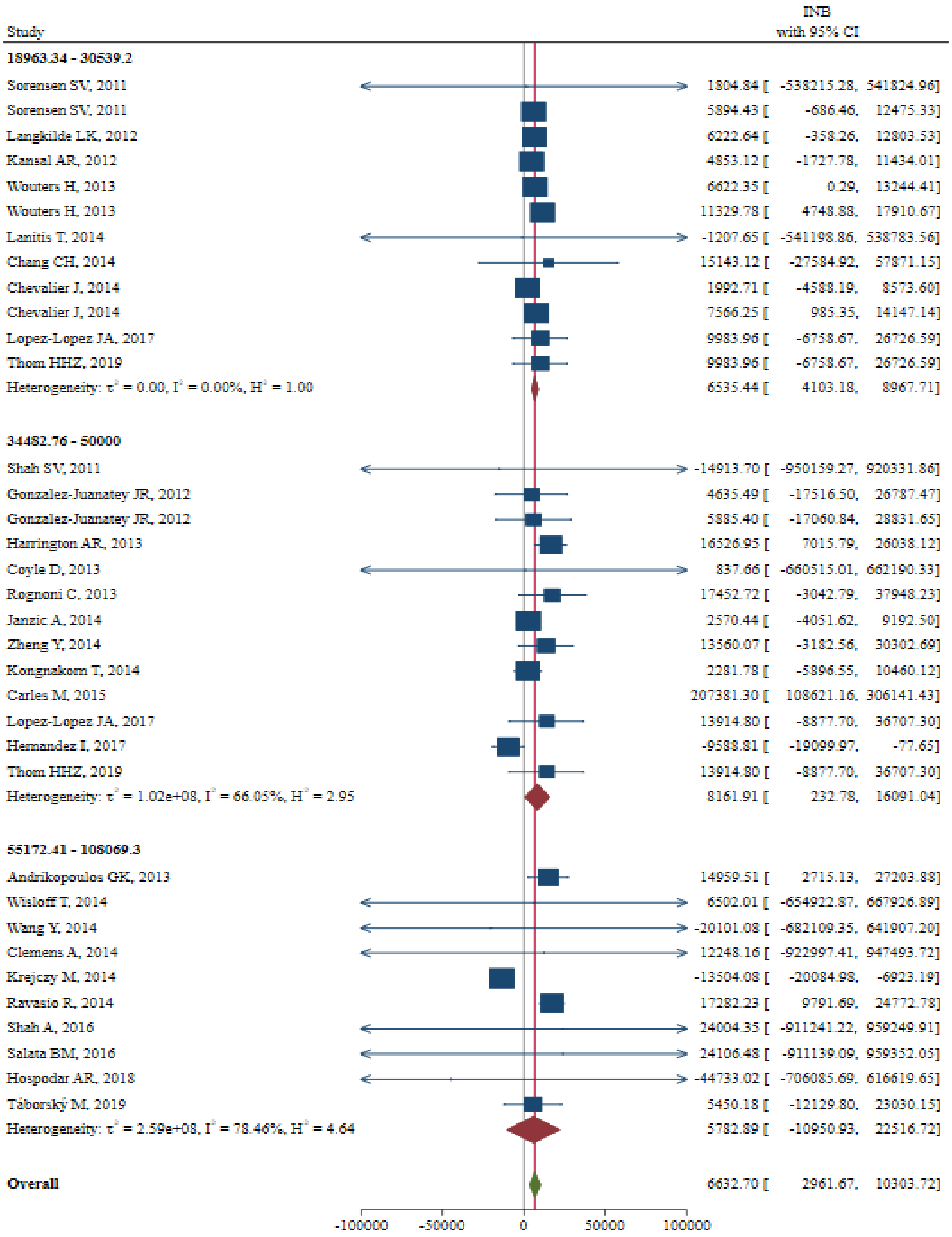

Abbreviations: INBs, Incremental Net Benefits; VKAs, Vitamin K Antagonists; HICs, High-Income Countries; TPP, Third-party payer perspective**.**

eFigure 4.6 Sub-group analysis by grant source of INB comparing Dabigatran with VKAs that estimated by Markov models with lifetime horizon and TPP in HICs. (Created by the authors)

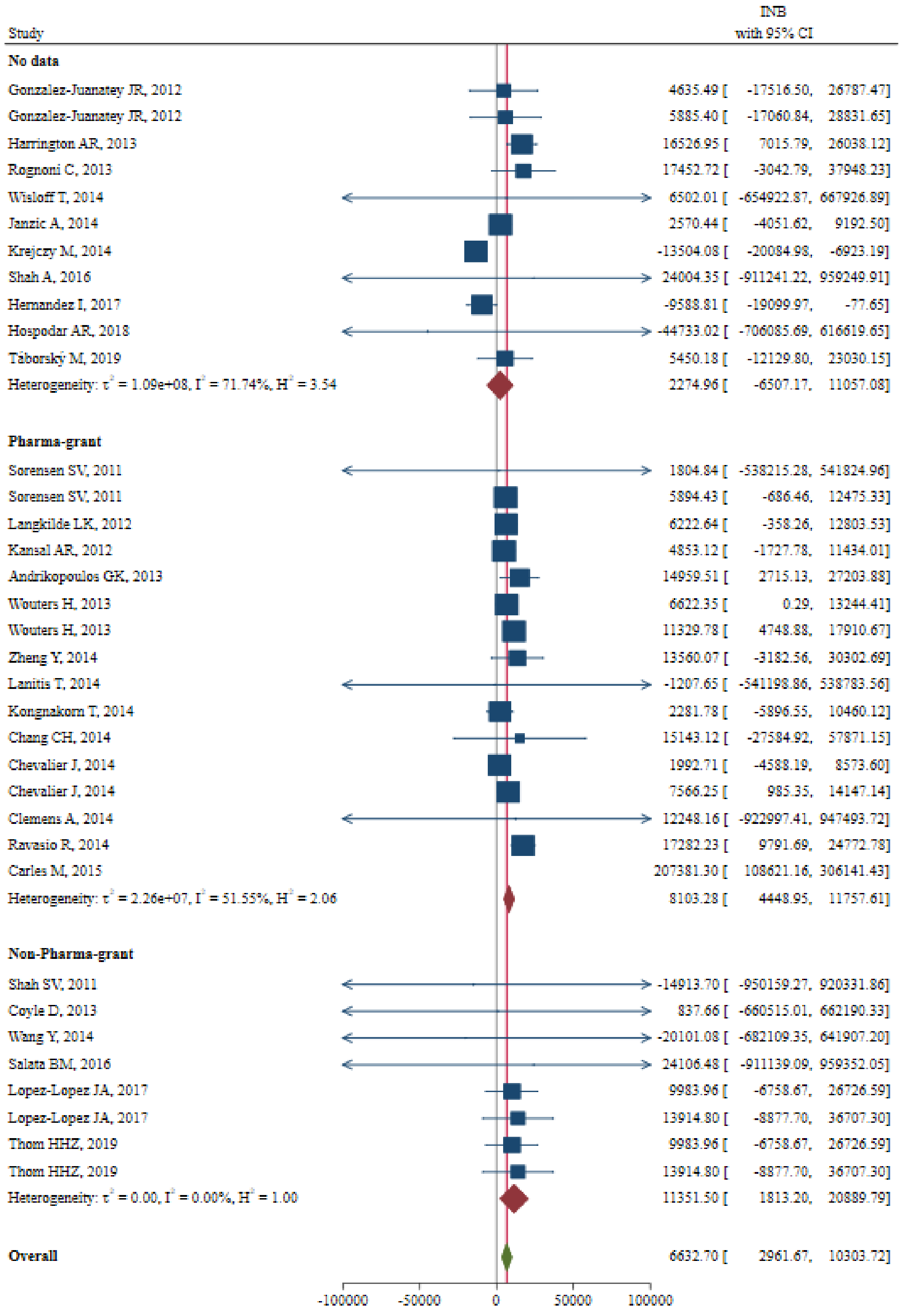

Abbreviations: INBs, Incremental Net Benefits; VKAs, Vitamin K Antagonists; HICs, High-Income Countries; TPP, Third-party payer perspective**.**

D) Publication Bias

Publication bias was assessed in each group of studies compared Dabigatran versus VKAs with similar in the level of country's income, Markov model, perspectives used and lifetime horizon, yielded the results:

High-income countries (HICs)

Assessment for the evidence of publication bias of those studies in HICs with Markov model, lifetime horizon and perspectives indicated a symmetry of the funnel plot (eFigure 4.7) as well as the Egger's test resulted coefficient=0.42, SE=0.27, p=0.130 in HICs with Markov model, lifetime horizon in TPP.

eFigure 4.7 Funnel plot comparing Dabigatran with VKAs that estimated by Markov models with lifetime horizon and TPP in HICs. (Created by the authors)

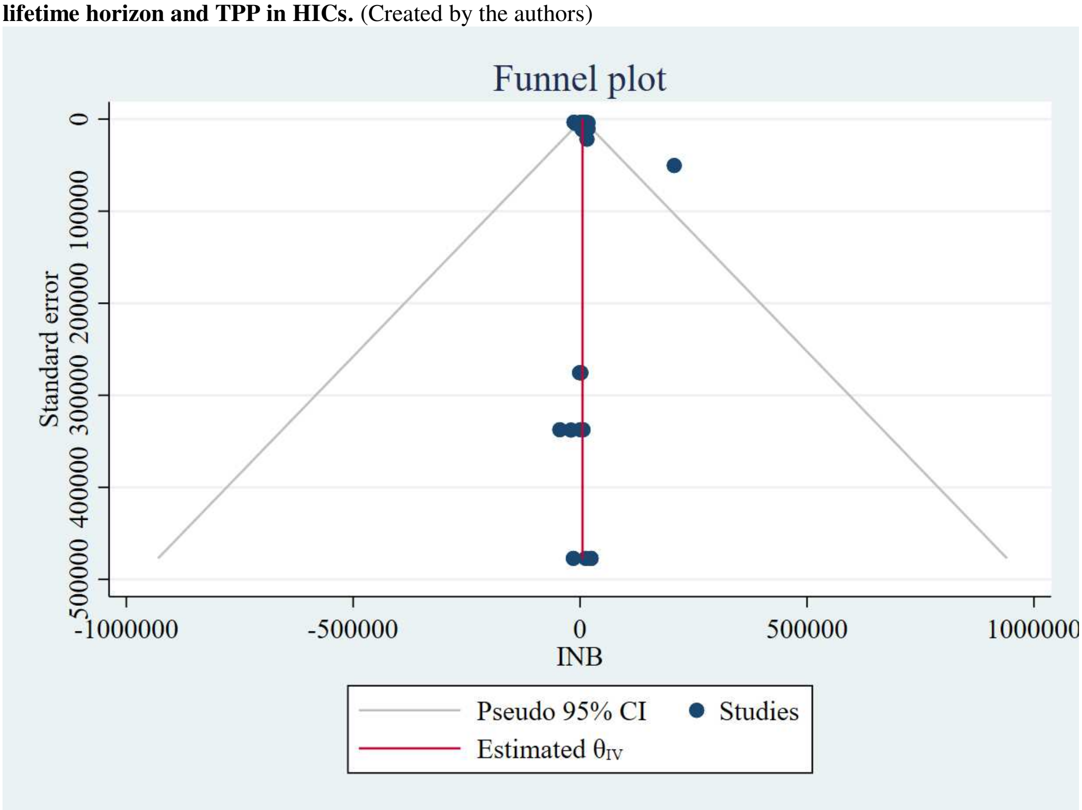

Abbreviations: VKAs, Vitamin K Antagonists; HICs, High-Income Countries; TPP, Third-party payer perspective**.**

Noviyani R, *et al. BMJ EBM* 2022; 27:215–223. doi: 10.1136/bmjebm-2020-111634

Appendix 5 Results of meta-analyses: Apixaban and Vitamin K Antagonists (VKAs)

A) Pooling INB

eFigure 5.1 Pooling INBs comparing Apixaban with VKAs in HICs estimated by Markov model, lifetime horizon and TPP. (Created by the authors)

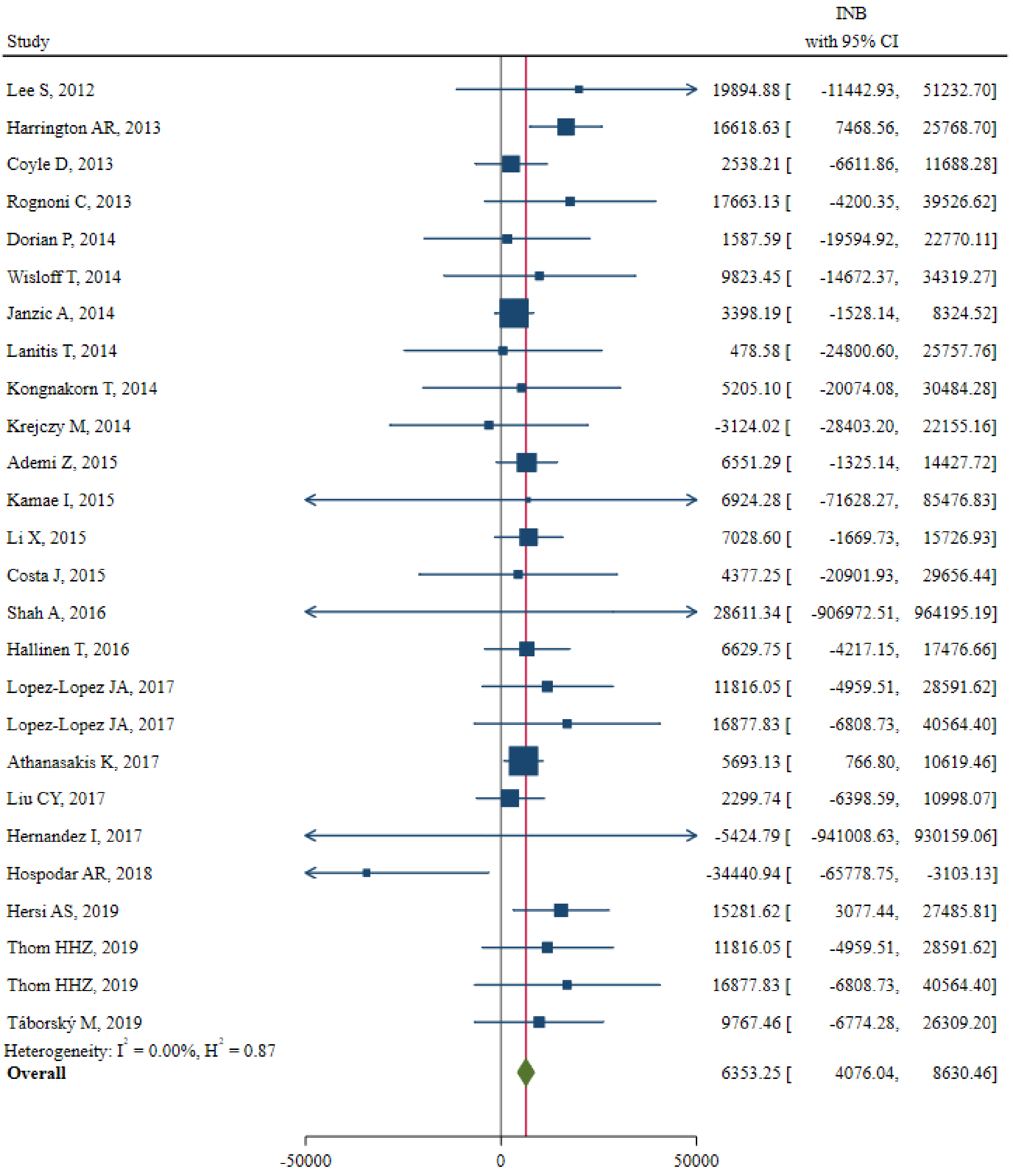

Abbreviations: INBs, Incremental Net Benefits; VKAs, Vitamin K Antagonists; HICs, High-Income Countries; TPP, Third-party payer perspective**.**

Noviyani R, *et al. BMJ EBM* 2022; 27:215–223. doi: 10.1136/bmjebm-2020-111634

eFigure 5.2 Pooling INBs comparing Apixaban with VKAs in HICs estimated by Markov model, lifetime horizon and SP. (Created by the authors)

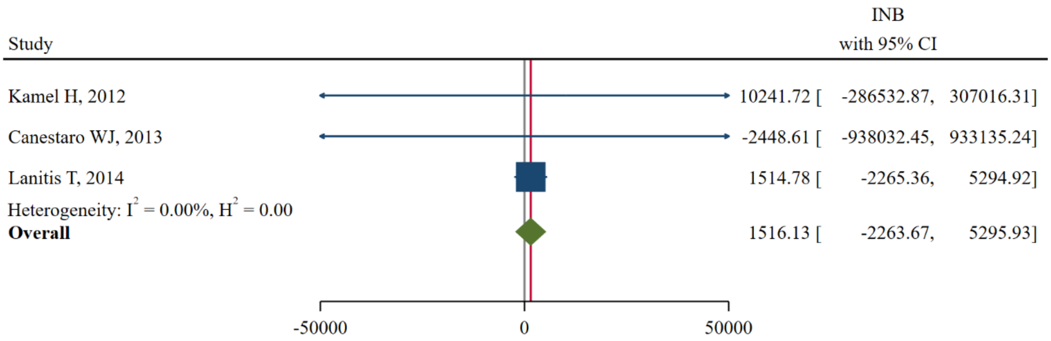

Abbreviations: INBs, Incremental Net Benefits; VKAs, Vitamin K Antagonists; HICs, High-Income Countries; SP, Societal perspective**.**

eFigure 5.3 Pooling INBs comparing Apixaban with VKAs in UMICs estimated by Markov model, lifetime horizon and TPP. (Created by the authors)

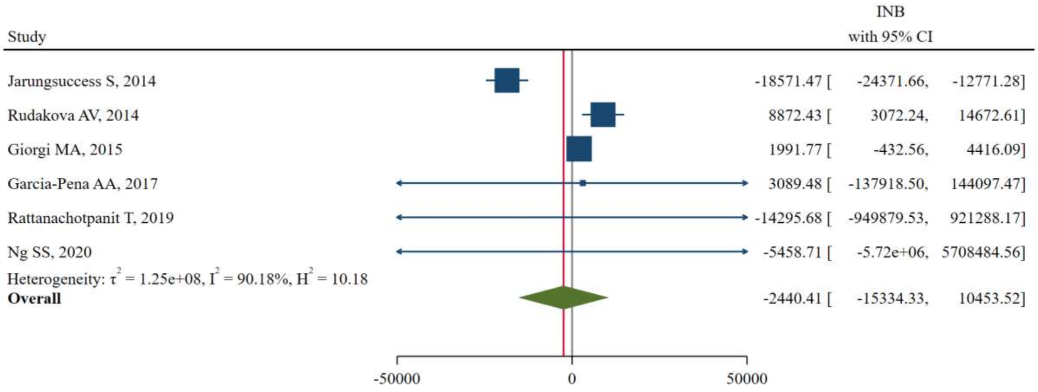

Abbreviations: INBs, Incremental Net Benefits; VKAs, Vitamin K Antagonists; UMICs, Upper Middle-Income Countries; TPP, Third-party payer perspective**.**

eFigure 5.4 Pooling INBs comparing Apixaban with VKAs in UMICs estimated by Markov model, lifetime horizon and SP. (Created by the authors)

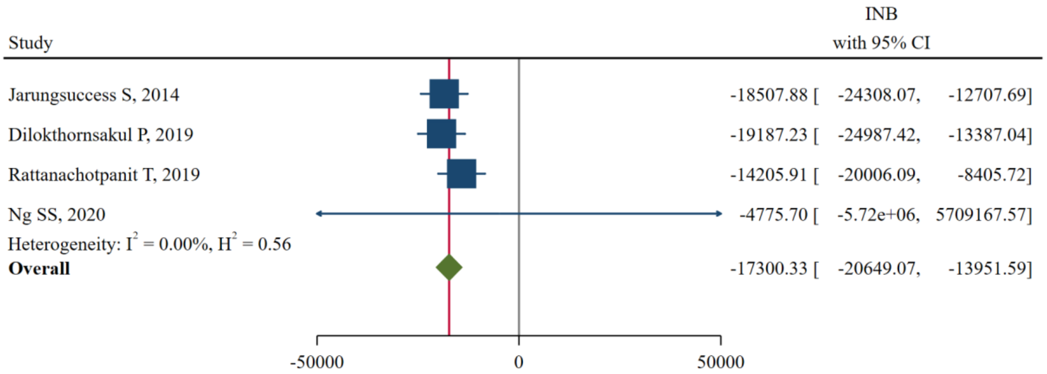

Abbreviations: INBs, Incremental Net Benefits; VKAs, Vitamin K Antagonists; UMICs, Upper Middle-Income Countries; SP, Societal perspective**.**

Noviyani R, *et al. BMJ EBM* 2022; 27:215–223. doi: 10.1136/bmjebm-2020-111634

B) Meta-regression analysis

eTable 5.1 Exploring source of heterogeneity using a meta-regression analysis. (Created by the authors)

**Factors**	**Coefficient**	**SE**	**P-value**	**I^2 (%)**
Apixaban vs VKAs in UMICs M TPP LT				
Model without factors	-2,440.41	6,578.654	0.726	90.18
WTP Threshold in USD				
16,389.31-770,414.2 vs 12424.11-16285.37	16,745.97	17,022.31	0.381	90.27
Discount cost				
≥3% vs <3%	22,522.73	3,985.208	0.005	14.76
Discount utility				
≥3% vs <3%	22,522.73	3,985.208	0.005	14.76
Clinical data source				
PL-Evidence synthesis vs PL	-22,494.21	3,896.37	0.004	13.11
Utility data source				
PL-Registry database vs PL	-11,856.68	477,527.1	0.981	92.15

Abbreviations: HICs, High-Income Countries; LT, lifetime; PL, Published Literature; SE, Standard Error; SP, Societal Perspective; TPP, Third-party payer perspective; UMICs, Upper Middle-Income Countries; VKAs, Vitamin K Antagonists, VS, versus; WTP, Willingness-to-Pay.

Noviyani R, *et al. BMJ EBM* 2022; 27:215–223. doi: 10.1136/bmjebm-2020-111634

C) Sub-group Analysis

eFigure 5.5 Sub-group analysis by discount cost of INB comparing Apixaban with VKAs that estimated by Markov models with lifetime horizon and TPP in UMICs. (Created by the authors)

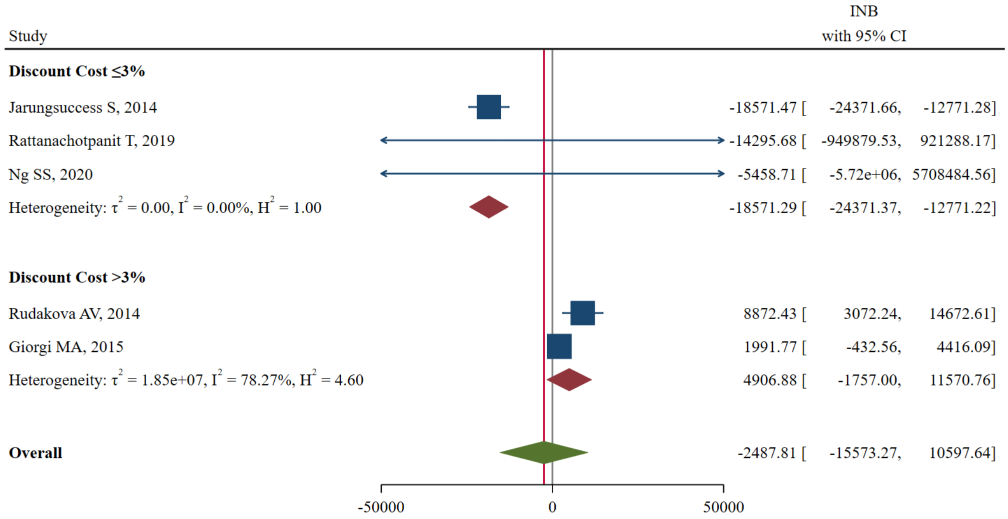

Abbreviations: INBs, Incremental Net Benefits; VKAs, Vitamin K Antagonists; UMICs, Upper Middle-Income Countries; TPP, Third-party payer perspective**.**

eFigure 5.6 Sub-group analysis by discount utility of INB comparing Apixaban with VKAs that estimated by Markov models with lifetime horizon and TPP in UMICs. (Created by the authors)

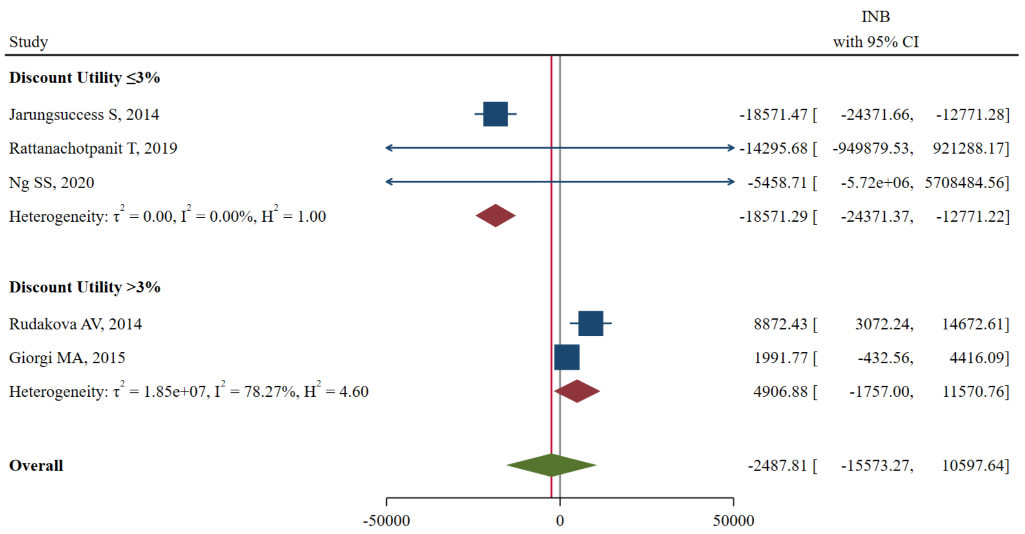

Abbreviations: INBs, Incremental Net Benefits; VKAs, Vitamin K Antagonists; UMICs, Upper Middle-Income Countries; TPP, Third-party payer perspective**.**

Noviyani R, *et al. BMJ EBM* 2022; 27:215–223. doi: 10.1136/bmjebm-2020-111634

eFigure 5.7 Sub-group analysis by clinical data source of INB comparing Apixaban with VKAs that estimated by Markov models with lifetime horizon and TPP in UMICs. (Created by the authors)

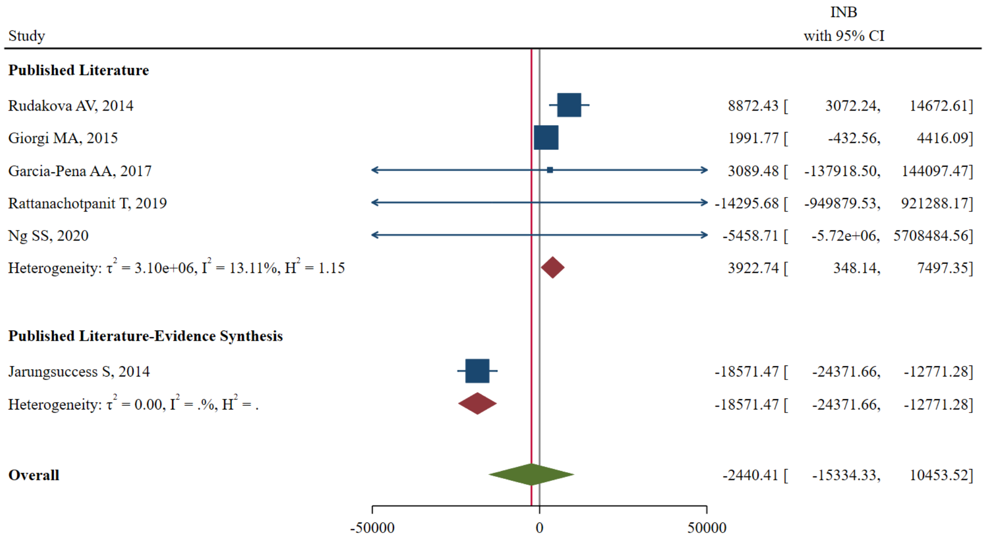

Abbreviations: INBs, Incremental Net Benefits; VKAs, Vitamin K Antagonists; UMICs, Upper Middle-Income Countries; TPP, Third-party payer perspective**.**

eFigure 5.8 Sub-group analysis by utility data source of INB comparing Apixaban with VKAs that estimated by Markov models with lifetime horizon and TPP in UMICs. (Created by the authors)

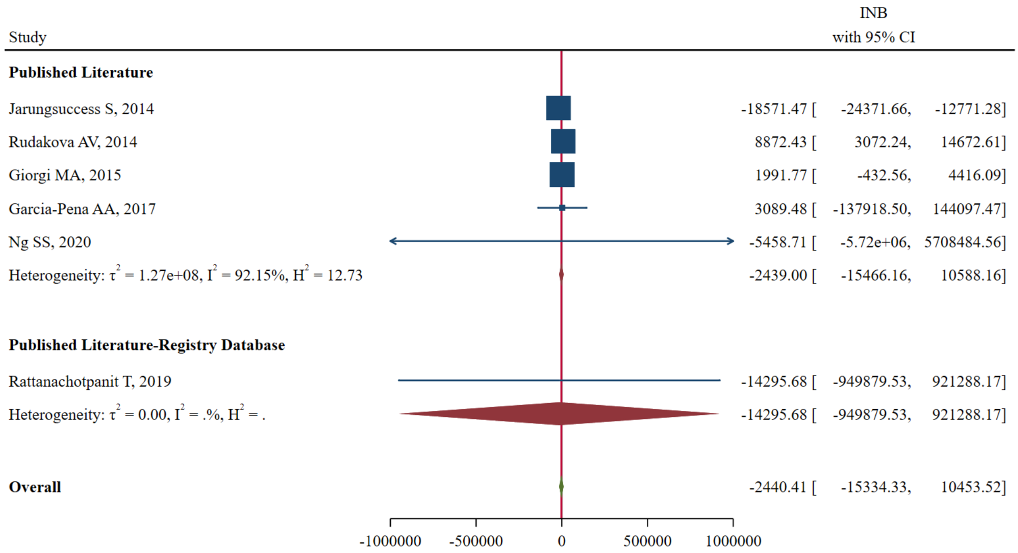

Abbreviations: INBs, Incremental Net Benefits; VKAs, Vitamin K Antagonists; UMICs, Upper Middle-Income Countries; TPP, Third-party payer perspective**.**

D) Publication Bias

Publication bias was assessed in each group of studies compared apixaban versus VKAs with similar in the level of country's income, Markov model, perspectives used and lifetime horizon, yielded the results:

High Income Countries (HICs)

Assessment for the evidence of the publication bias of those studies in HICs with Markov, lifetime and perspectives indicated a symmetry of the funnel plot (eFigure 5.9) as well as the Egger's test resulted coefficient= 0.20, SE=0.33, p=0.538 in HICs with Markov lifetime with TPP.

eFigure 5.9 Funnel plot comparing Apixaban with VKAs that estimated by Markov models with lifetime horizon and TPP in HICs. (Created by the authors)

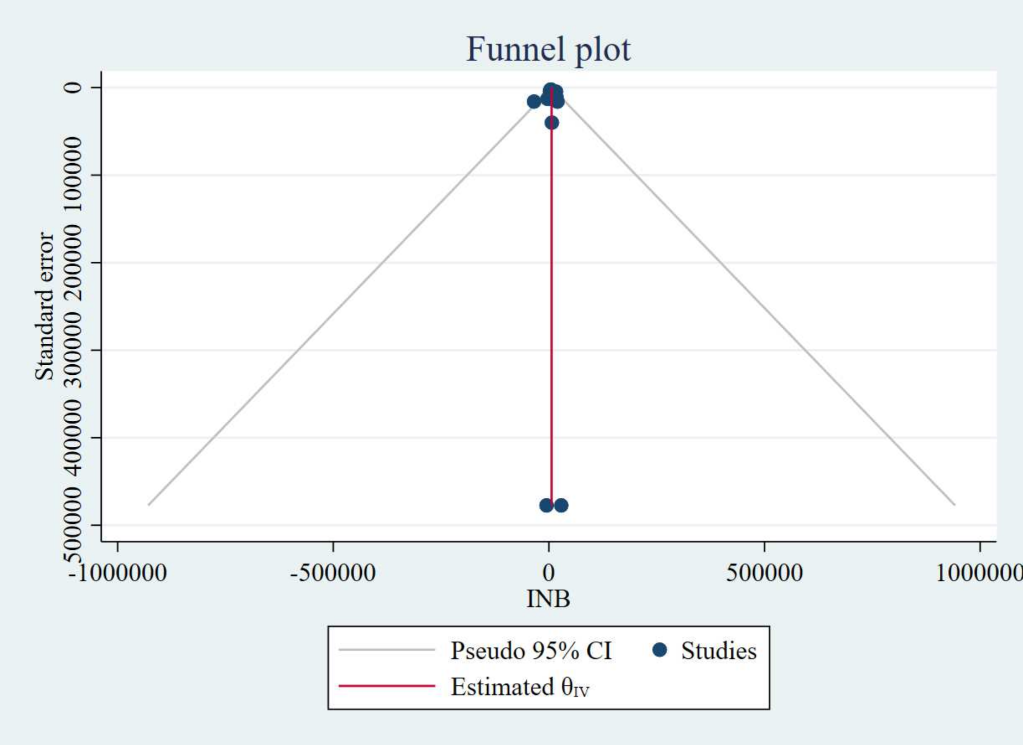

Abbreviations: VKAs, Vitamin K Antagonists; HICs, High-Income Countries; TPP, Third-party payer perspective**.**

Appendix 6 Results of meta-analyses: Rivaroxaban and Vitamin K Antagonists (VKAs)

A) Pooling INB

eFigure 6.1 Pooling INBs comparing Rivaroxaban with VKAs in HICs estimated by Markov model, lifetime horizon, and TPP. (Created by the authors)

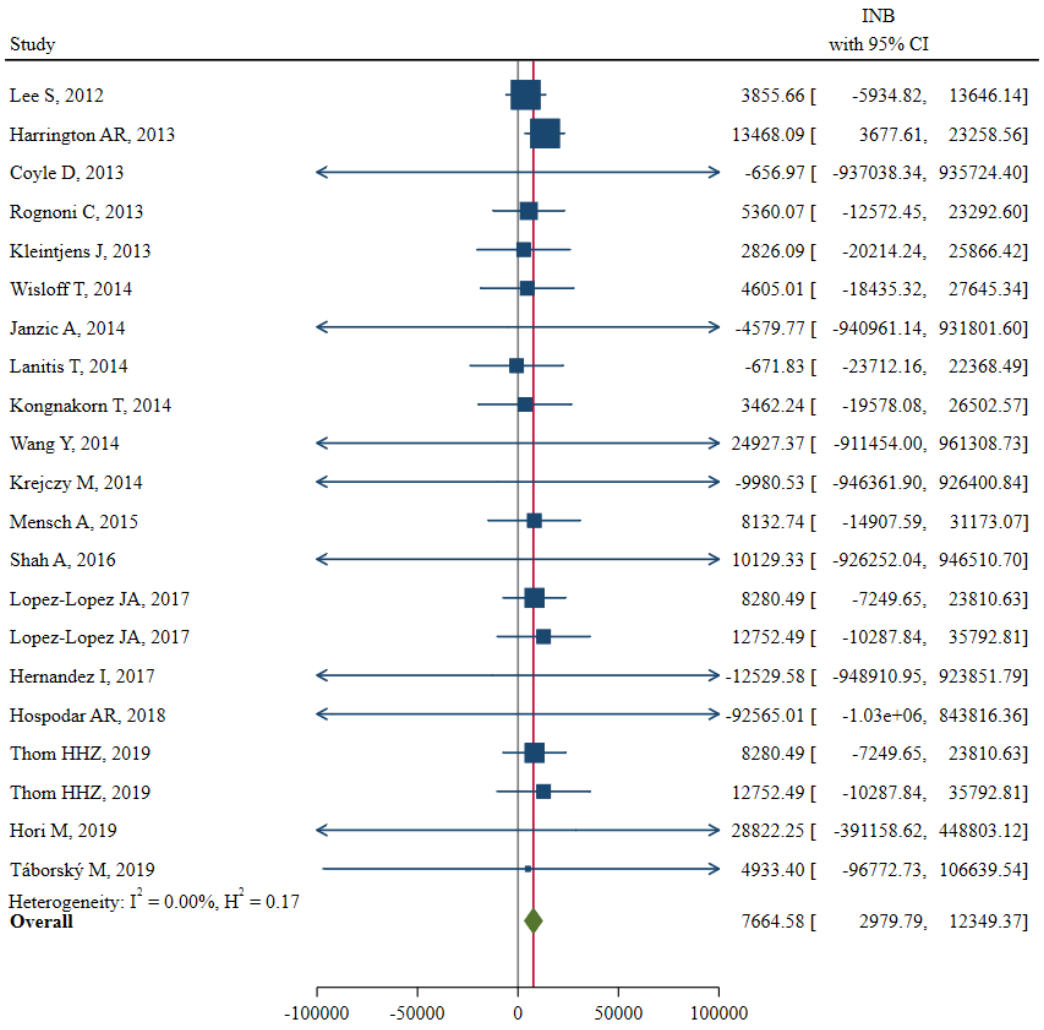

Abbreviations: INBs, Incremental Net Benefits; VKAs, Vitamin K Antagonists; HICs, High-Income Countries; TPP, Third-party payer perspective**.**

Noviyani R, *et al. BMJ EBM* 2022; 27:215–223. doi: 10.1136/bmjebm-2020-111634

eFigure 6.2 Pooling INBs comparing Rivaroxaban with VKAs in HICs estimated by Markov model, lifetime horizon, and SP. (Created by the authors)

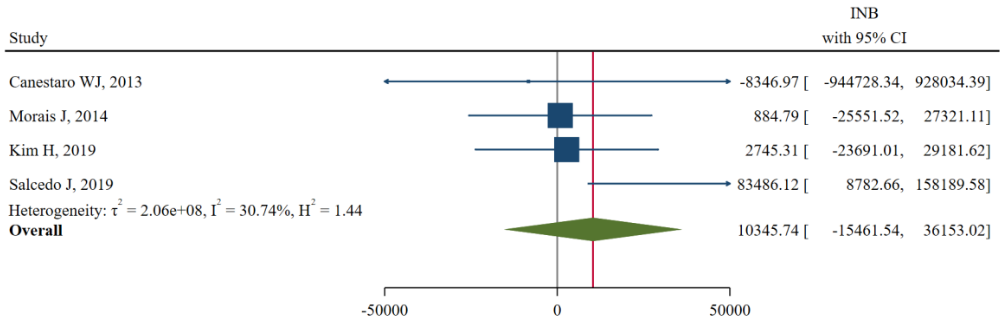

Abbreviations: INBs, Incremental Net Benefits; VKAs, Vitamin K Antagonists; HICs, High-Income Countries; SP, Societal perspective**.**

eFigure 6.3 Pooling INBs comparing Rivaroxaban with VKAs in UMICs estimated by Markov model, lifetime horizon, and TPP. (Created by the authors)

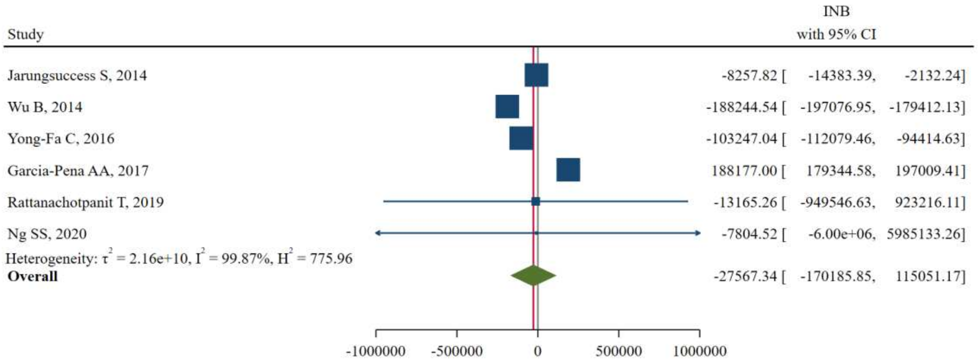

Abbreviations: INBs, Incremental Net Benefits; VKAs, Vitamin K Antagonists; UMICs, Upper Middle-Income Countries; TPP, Third-party payer perspective.

eFigure 6.4 Pooling INBs comparing Rivaroxaban with VKAs in UMICs estimated by Markov model, lifetime horizon, and SP. (Created by the authors)

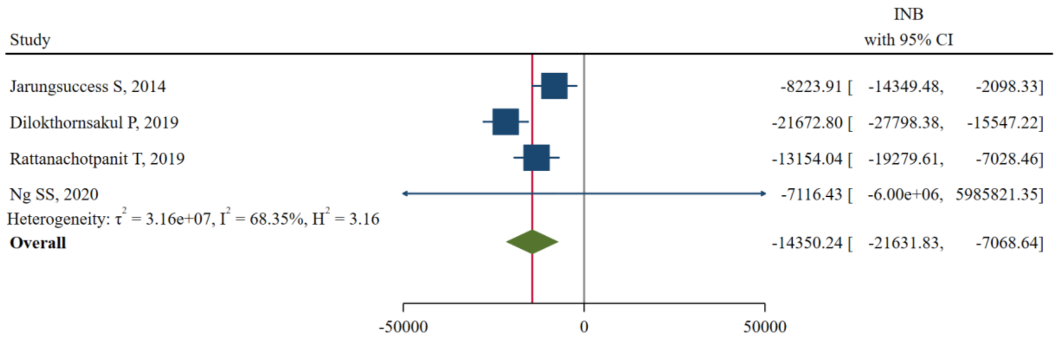

Abbreviations: INBs, Incremental Net Benefits; VKAs, Vitamin K Antagonists; UMICs, Upper Middle-Income Countries; SP, Societal perspective**.**

B) Meta-regression analysis

eTable 6.1 Exploring source of heterogeneity by a meta-regression analysis. (Created by the authors)

**Factors**	**Coefficient**	**SE**	**P-value**	**I^2 (%)**
Rivaroxaban vs VKAs in UMICs M TPP LT				
Model without factor	-27,567.34	72,765.88	0.720	99.87
WTP Threshold				
34,210.87-770,414.2 vs 12959.46-16389.31	-25,560.01	216,442.7	0.912	99.90
Clinical data source				
PL-Evidence synthesis vs PL	25,160.73	226,510.4	0.917	99.90
Utility data source				
PL-Registry database vs PL	14,713.85	505,271.6	0.978	99.90

Abbreviations: HICs, High-Income Countries; LT, lifetime; PL, Published Literature; SE, Standard Error; SP, Societal Perspective; TPP, Third-party payer perspective; UMICs, Upper Middle-Income Countries; VKAs, Vitamin K Antagonists, VS, versus; WTP, Willingness-to-Pay.

C) Publication Bias

Publication bias was assessed in each group of studies compared rivaroxaban versus VKAs with similar in the level of country's income, Markov model, perspectives used and lifetime horizon, yielded the results:

High-Income Countries (HICs)

Assessment for the evidence of publication bias of those studies in HICs with Markov, lifetime and perspectives indicated a symmetry of the funnel plot as well as the (eFigure 6.5) as well as the Egger's test resulted coefficient=-0.08, SE=0.32, p=0.805 in HICs and Markov model, lifetime horizon with TPP.

eFigure 6.5 Funnel plot comparing Rivaroxaban with VKAs that estimated by Markov models with lifetime horizon and TPP in HICs. (Created by the authors)

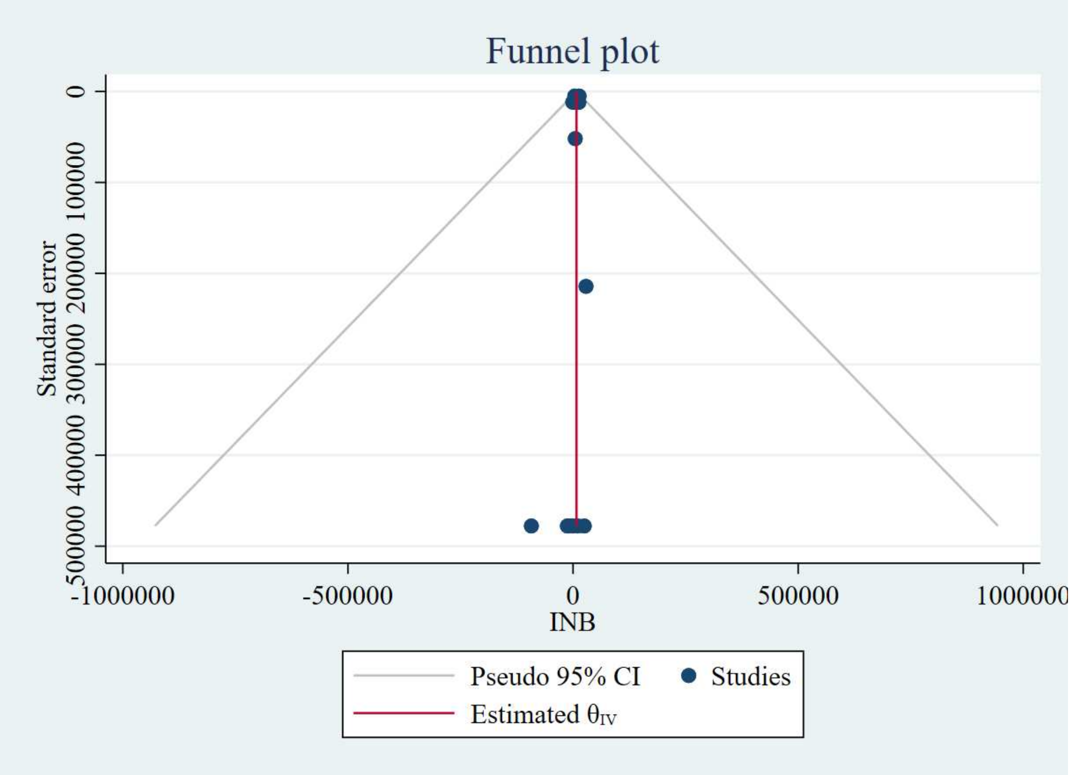

Abbreviations: VKAs, Vitamin K Antagonists; HICs, High-Income Countries; TPP, Third-party payer perspective**.**

Appendix 7 Results of meta-analyses: Edoxaban and Vitamin K Antagonists (VKAs)

A) Pooling INB

eFigure 7.1 Pooling INBs comparing Edoxaban with VKAs in HICs estimated by Markov model, lifetime horizon, and TPP. (Created by the authors)

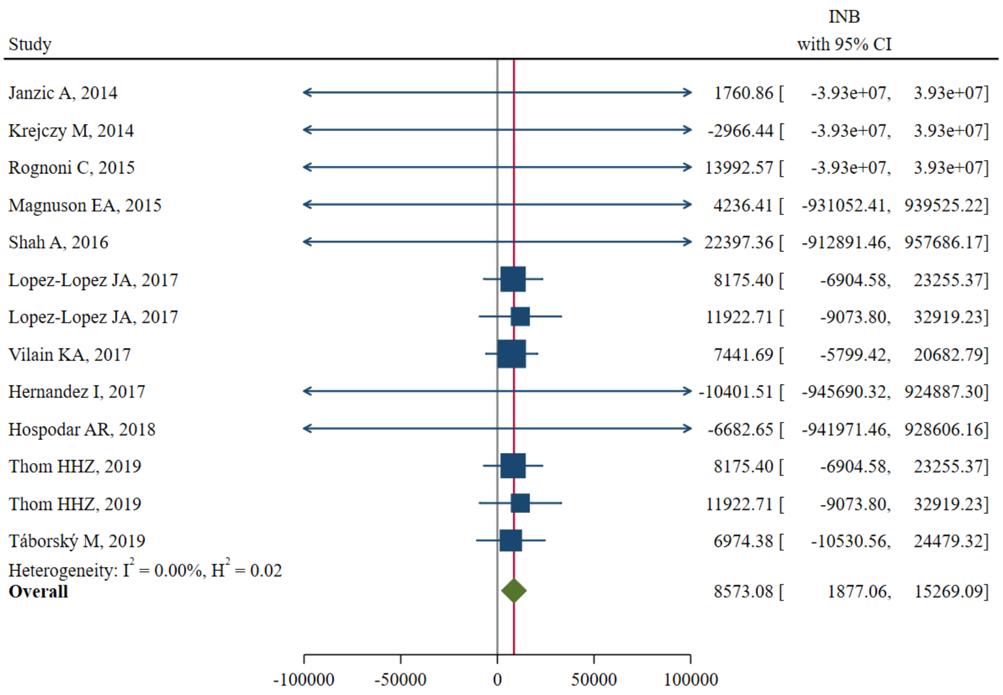

Abbreviations: INBs, Incremental Net Benefits; VKAs, Vitamin K Antagonists; HICs, High-Income Countries; TPP, Third-party payer perspective**.**

eFigure 7.2 Pooling INBs comparing Edoxaban with VKAs in UMICs estimated by Markov model, lifetime horizon, and TPP. (Created by the authors)

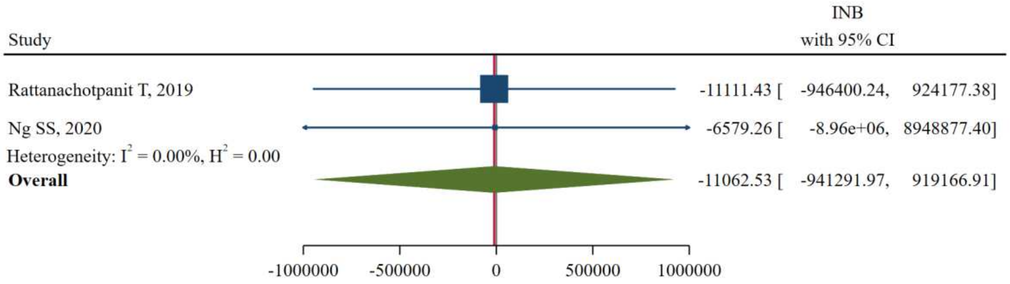

Abbreviations: INBs, Incremental Net Benefits; VKAs, Vitamin K Antagonists; UMICs, Upper Middle-Income Countries; TPP, Third-party payer perspective.

Noviyani R, *et al. BMJ EBM* 2022; 27:215–223. doi: 10.1136/bmjebm-2020-111634

eFigure 7.3 Pooling INBs comparing Edoxaban with VKAs in UMICs estimated by Markov model, lifetime horizon, and SP. (Created by the authors)

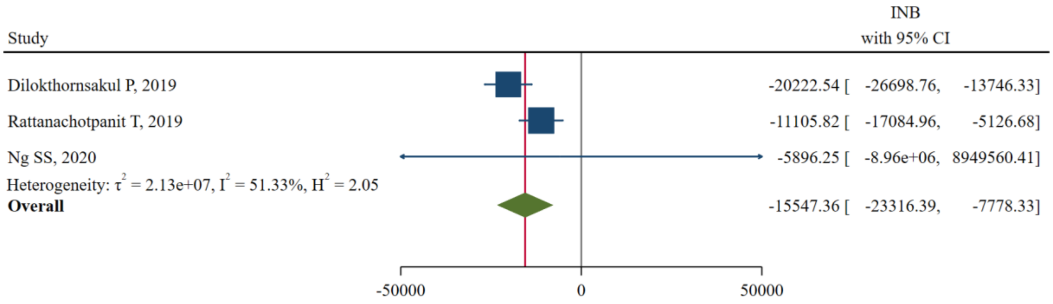

Abbreviations: INBs, Incremental Net Benefits; VKAs, Vitamin K Antagonists; UMICs, Upper Middle-Income Countries; SP, Societal perspective**.**

References:

1. Crespo C, Monleon A, Diaz W, et al. Comparative efficiency research (COMER): meta-analysis of cost-effectiveness studies. *BMC Med Res Methodol* 2014;14:139. doi: 10.1186/1471-2288-14-139 [published Online First: 2014/12/24]
2. Briggs A CK, Sculpher M. Decision modelling for health economic evaluation: Oxford University Press 2006:121.
3. Rohatgi A. WebPlotDigitizer [Available from: https://automeris.io/WebPlotDigitizer/download.html (last accessed July 20th, 2019).
4. DerSimonian R, Laird N. Meta-analysis in clinical trials revisited. *Contemporary Clinical Trials* 2015;45(Pt A):139-45. doi: 10.1016/j.cct.2015.09.002 [published Online First: 2015/09/08]
5. Abdullaev SP, Mirzaev KB, Sychev DA. [Comparative clinical and economic evaluation of pharmacogenetic testing application for dabigatran in patients with atrial fibrillation]. *Ter Arkh* 2019;91(8):22-27. doi: 10.26442/00403660.2019.08.000379 [published Online First: 2020/07/01]
6. Belousov YB, Mareev VY, Yavelov IS, et al. Pharmacoeconomic evaluation of dabigatran vs warfarin in cardiovascular events prevention in patients with non-valvular atrial fibrillation. *Rational Pharmacotherapy in Cardiology* 2012;8(1):37-44. doi: 10.20996/1819-6446-2012-8-1-37-44
7. Bonet Pla A, Gosalbes Soler V, Ridao-Lopez M, et al. [Dabigatran versus acenocumarol for the prevention of stroke in atrial fibrillation: budget impact analysis in one health department in Spain]. *Rev Esp Salud Publica* 2013;87(4):331-42. doi: 10.4321/s1135-57272013000400004 [published Online First: 2013/10/09]
8. Kansal AR, Zheng Y, Pokora T, et al. Cost-effectiveness of new oral anticoagulants in the prevention of stroke in patients with atrial fibrillation. *Best Pract Res Clin Haematol* 2013;26(2):225-37. doi: 10.1016/j.beha.2013.07.012 [published Online First: 2013/08/21]
9. Koretsune Y. Cost-effectiveness analysis of edoxaban for patients with non-valvular atrial fibrillation. *Therapeutic Research* 2018;39(5):419-39.
10. Monreal-Bosch M, Soulard S, Crespo C, et al. [Comparison of the cost-utility of direct oral anticoagulants for the prevention of stroke in patients with atrial fibrillation in Spain]. *Rev Neurol* 2017;64(6):247-56. [published Online First: 2017/03/09]
11. Nedogoda SV, Barykina IN, Salasiuk AS, et al. Clinical and economical comparison of rivaroxaban and apixaban use in patients with non-valvular atrial fibrillation. *Rational Pharmacotherapy in Cardiology* 2017;13(1):45-50. doi: 10.20996/1819-6446-2017-13-1-45-50
12. Rudakova AV, Tatarskii BA. [Cost-effectiveness of apixaban compared to other new oral anticoagulants in patients with non-valvular atrial fibrillation]. *Kardiologiia* 2014;54(7):43-52. doi: 10.18565/cardio.2014.7.43-52 [published Online First: 2014/09/02]
13. Sorensen SV, Peng S, Monz BU, et al. A comparative analysis of models used to evaluate the cost-effectiveness of dabigatran versus warfarin for the prevention of stroke in atrial fibrillation. *Pharmacoeconomics* 2013;31(7):589-604. doi: 10.1007/s40273-013-0035-8 [published Online First: 2013/04/26]
14. Uetsuka Y. Cost-effectiveness of oral anticoagulant in patients with atrial fibrillation. *Japanese Journal of Clinical Pharmacology and Therapeutics* 2011;42(5):321-32. doi: 10.3999/jscpt.42.321
15. Vestergaard AS, Ehlers LH. A Health Economic Evaluation of Stroke Prevention in Atrial Fibrillation: Guideline Adherence Versus the Observed Treatment Strategy Prior to 2012 in Denmark. *Pharmacoeconomics* 2015;33(9):967-79. doi: 10.1007/s40273-015-0281-z
16. You JHS, Tsui KKN, Wong RSM, et al. Cost-effectiveness of Dabigatran versus genotype-guided management of Warfarin therapy for stroke prevention in patients with Atrial fibrillation. *PLoS One* 2012;7(6) doi: 10.1371/journal.pone.0039640
17. You JH. Universal versus genotype-guided use of direct oral anticoagulants in atrial fibrillation patients: A decision analysis. *Pharmacogenomics* 2015;16(10):1089-100. doi: 10.2217/pgs.15.64

18. You JHS. Pharmacogenetic-guided selection of warfarin versus novel oral anticoagulants for stroke prevention in patients with atrial fibrillation: A cost-effectiveness analysis. *Pharmacogenet Genomics* 2014;24(1):6-14. doi: 10.1097/FPC.0000000000000014
19. Pink J, Lane S, Pirmohamed M, et al. Dabigatran etexilate versus warfarin in management of non-valvular atrial fibrillation in UK context: quantitative benefit-harm and economic analyses. *Bmj* 2011;343:d6333. doi: 10.1136/bmj.d6333 [published Online First: 2011/11/02]
20. Dilokthornsakul P, Nathisuwan S, Krittayaphong R, et al. Cost-Effectiveness Analysis of Non-Vitamin K Antagonist Oral Anticoagulants Versus Warfarin in Thai Patients With Non-Valvular Atrial Fibrillation. *Heart Lung Circ* 2020:390-400. doi: 10.1016/j.hlc.2019.02.187 [published Online First: 2019/04/20]
21. Harrington AR, Armstrong EP, Nolan PE, Jr., et al. Cost-effectiveness of apixaban, dabigatran, rivaroxaban, and warfarin for stroke prevention in atrial fibrillation. *Stroke* 2013;44(6):1676-81. doi: 10.1161/strokeaha.111.000402 [published Online First: 2013/04/04]
22. Lopez-Lopez JA, Sterne JAC, Thom HHZ, et al. Oral anticoagulants for prevention of stroke in atrial fibrillation: systematic review, network meta-analysis, and cost effectiveness analysis. *Bmj* 2017;359:j5058. doi: 10.1136/bmj.j5058 [published Online First: 2017/12/01]
23. Verhoef TI, Redekop WK, Hasrat F, et al. Cost effectiveness of new oral anticoagulants for stroke prevention in patients with atrial fibrillation in two different European healthcare settings. *Am J Cardiovasc Drugs* 2014;14(6):451-62. doi: 10.1007/s40256-014-0092-1 [published Online First: 2014/10/19]
24. Stevanovic J, Pompen M, Le HH, et al. Economic evaluation of apixaban for the prevention of stroke in non-valvular atrial fibrillation in the Netherlands. *PLoS One* 2014;9(8):e103974. doi: 10.1371/journal.pone.0103974 [published Online First: 2014/08/06]
25. Ademi Z, Pasupathi K, Liew D. Cost-effectiveness of apixaban compared to warfarin in the management of atrial fibrillation in Australia. *Eur J Prev Cardiol* 2015;22(3):344-53. doi: 10.1177/2047487313514019 [published Online First: 2013/11/28]
26. Andrikopoulos GK, Fragoulakis V, Maniadakis N. Economic evaluation of dabigatran etexilate in the management of atrial fibrillation in Greece. *Hellenic J Cardiol* 2013;54(4):289-300. [published Online First: 2013/08/06]
27. Shah A, Shewale A, Hayes CJ, et al. Cost-Effectiveness of Oral Anticoagulants for Ischemic Stroke Prophylaxis Among Nonvalvular Atrial Fibrillation Patients. *Stroke* 2016;47(6):1555-61. doi: 10.1161/strokeaha.115.012325 [published Online First: 2016/04/23]
28. Kamae I, Hashimoto Y, Koretsune Y, et al. Cost-effectiveness Analysis of Apixaban against Warfarin for Stroke Prevention in Patients with Nonvalvular Atrial Fibrillation in Japan. *Clin Ther* 2015;37(12):2837-51. doi: 10.1016/j.clinthera.2015.10.007 [published Online First: 2015/11/27]
29. Lip GY, Kongnakorn T, Phatak H, et al. Cost-effectiveness of apixaban versus other new oral anticoagulants for stroke prevention in atrial fibrillation. *Clin Ther* 2014;36(2):192-210.e20. doi: 10.1016/j.clinthera.2013.12.011 [published Online First: 2014/02/11]
30. Jarungsuccess S, Taerakun S. Cost-utility analysis of oral anticoagulants for nonvalvular atrial fibrillation patients at the police general hospital, Bangkok, Thailand. *Clin Ther* 2014;36(10):1389-94.e4. doi: 10.1016/j.clinthera.2014.08.016 [published Online First: 2014/10/01]
31. Athanasakis K, Karampli E, Tsounis D, et al. Cost-effectiveness of apixaban vs. other new oral anticoagulants for the prevention of stroke: an analysis on patients with non-valvular atrial fibrillation in the Greek healthcare setting. *Clin Drug Investig* 2015;35(11):693-705. doi: 10.1007/s40261-015-0321-7 [published Online First: 2015/09/20]
32. Coyle D, Coyle K, Cameron C, et al. Cost-effectiveness of new oral anticoagulants compared with warfarin in preventing stroke and other cardiovascular events in patients with atrial fibrillation. *Value Health* 2013;16(4):498-506. doi: 10.1016/j.jval.2013.01.009 [published Online First: 2013/06/26]
33. Pletscher M, Plessow R, Eichler K, et al. Cost-effectiveness of dabigatran for stroke prevention in atrial fibrillation in Switzerland. *Swiss Med Wkly* 2013;143:w13732. doi: 10.4414/smw.2013.13732 [published Online First: 2013/01/10]

34. Miller JD, Ye X, Lenhart GM, et al. Cost-effectiveness of edoxaban versus rivaroxaban for stroke prevention in patients with nonvalvular atrial fibrillation (NVAF) in the US. *ClinicoEconomics and outcomes research : CEOR* 2016;8:215-26. doi: 10.2147/ceor.S98888 [published Online First: 2016/06/11]
35. Magnuson EA, Vilain K, Wang K, et al. Cost-effectiveness of edoxaban vs warfarin in patients with atrial fibrillation based on results of the ENGAGE AF-TIMI 48 trial. *Am Heart J* 2015;170(6):1140-50. doi: 10.1016/j.ahj.2015.09.011 [published Online First: 2015/12/19]
36. Athanasakis K, Boubouchairopoulou N, Karampli E, et al. Cost Effectiveness of Apixaban versus Warfarin or Aspirin for Stroke Prevention in Patients with Atrial Fibrillation: A Greek Perspective. *Am J Cardiovasc Drugs* 2017;17(2):123-33. doi: 10.1007/s40256-016-0204-1 [published Online First: 2016/11/25]
37. Kamel H, Easton JD, Johnston SC, et al. Cost-effectiveness of apixaban vs warfarin for secondary stroke prevention in atrial fibrillation. *Neurology* 2012;79(14):1428-34. doi: 10.1212/WNL.0b013e31826d5fe8 [published Online First: 2012/09/21]
38. Pink J, Pirmohamed M, Lane S, et al. Cost-effectiveness of pharmacogenetics-guided warfarin therapy vs. alternative anticoagulation in atrial fibrillation. *Clin Pharmacol Ther* 2014;95(2):199-207. doi: 10.1038/clpt.2013.190 [published Online First: 2013/09/27]
39. Dorian P, Kongnakorn T, Phatak H, et al. Cost-effectiveness of apixaban vs. current standard of care for stroke prevention in patients with atrial fibrillation. *Eur Heart J* 2014;35(28):1897-906. doi: 10.1093/eurheartj/ehu006 [published Online First: 2014/02/12]
40. Lanitis T, Kongnakorn T, Jacobson L, et al. Cost-effectiveness of apixaban versus warfarin and aspirin in Sweden for stroke prevention in patients with atrial fibrillation. *Thromb Res* 2014;134(2):278-87. doi: 10.1016/j.thromres.2014.05.027 [published Online First: 2014/06/18]
41. Canestaro WJ, Patrick AR, Avorn J, et al. Cost-effectiveness of oral anticoagulants for treatment of atrial fibrillation. *Circ Cardiovasc Qual Outcomes* 2013;6(6):724-31. doi: 10.1161/circoutcomes.113.000661 [published Online First: 2013/11/14]
42. Wisloff T, Hagen G, Klemp M. Economic evaluation of warfarin, dabigatran, rivaroxaban, and apixaban for stroke prevention in atrial fibrillation. *Pharmacoeconomics* 2014;32(6):601-12. doi: 10.1007/s40273-014-0152-z [published Online First: 2014/04/10]
43. Janzic A, Kos M. Cost effectiveness of novel oral anticoagulants for stroke prevention in atrial fibrillation depending on the quality of warfarin anticoagulation control. *Pharmacoeconomics* 2015;33(4):395-408. doi: 10.1007/s40273-014-0246-7 [published Online First: 2014/12/17]
44. Zheng Y, Sorensen SV, Gonschior AK, et al. Comparison of the cost-effectiveness of new oral anticoagulants for the prevention of stroke and systemic embolism in atrial fibrillation in a UK setting. *Clin Ther* 2014;36(12):2015-28.e2. doi: 10.1016/j.clinthera.2014.09.015 [published Online First: 2014/12/03]
45. Baron Esquivias G, Escolar Albaladejo G, Zamorano JL, et al. Cost-effectiveness Analysis Comparing Apixaban and Acenocoumarol in the Prevention of Stroke in Patients With Nonvalvular Atrial Fibrillation in Spain. *Rev Esp Cardiol (Engl Ed)* 2015;68(8):680-90. doi: 10.1016/j.rec.2014.08.010 [published Online First: 2014/12/17]
46. Giorgi MA, Caroli C, Giglio ND, et al. Estimation of the cost-effectiveness of apixaban versus vitamin K antagonists in the management of atrial fibrillation in Argentina. *Health Econ Rev* 2015;5(1):52. doi: 10.1186/s13561-015-0052-8 [published Online First: 2015/06/27]
47. Pradelli L, Calandriello M, Di VR, et al. Cost Effectiveness Analysis Of Apixaban Versus Other Noacs For The Prevention Of Stroke In Italian Non-Valvular Atrial Fibrillation Patients. *Value Health* 2014;17(7):A487-8. doi: 10.1016/j.jval.2014.08.1432 [published Online First: 2014/11/01]
48. Li X, Tse VC, Lau WC, et al. Cost-Effectiveness of Apixaban versus Warfarin in Chinese Patients with Non-Valvular Atrial Fibrillation: A Real-Life and Modelling Analyses. *PLoS One* 2016;11(6):e0157129. doi: 10.1371/journal.pone.0157129 [published Online First: 2016/07/01]
49. Lanitis T, Cotte FE, Gaudin AF, et al. Stroke prevention in patients with atrial fibrillation in France: comparative cost-effectiveness of new oral anticoagulants (apixaban, dabigatran, and

rivaroxaban), warfarin, and aspirin. *J Med Econ* 2014;17(8):587-98. doi: 10.3111/13696998.2014.923891 [published Online First: 2014/05/17]
50. Krejczy M, Harenberg J, Wehling M, et al. Cost-effectiveness of anticoagulation in patients with nonvalvular atrial fibrillation with edoxaban compared to warfarin in Germany. *Biomed Res Int* 2015;2015:876923. doi: 10.1155/2015/876923 [published Online First: 2015/04/09]
51. Lee S, Mullin R, Blazawski J, et al. Cost-effectiveness of apixaban compared with warfarin for stroke prevention in atrial fibrillation. *PLoS One* 2012;7(10):e47473. doi: 10.1371/journal.pone.0047473 [published Online First: 2012/10/12]
52. Rognoni C, Marchetti M, Quaglini S, et al. Apixaban, dabigatran, and rivaroxaban versus warfarin for stroke prevention in non-valvular atrial fibrillation: a cost-effectiveness analysis. *Clin Drug Investig* 2014;34(1):9-17. doi: 10.1007/s40261-013-0144-3 [published Online First: 2013/10/19]
53. Kongnakorn T, Lanitis T, Annemans L, et al. Stroke and systemic embolism prevention in patients with atrial fibrillation in Belgium: comparative cost effectiveness of new oral anticoagulants and warfarin. *Clin Drug Investig* 2015;35(2):109-19. doi: 10.1007/s40261-014-0253-7 [published Online First: 2014/12/17]
54. Mensch A, Stock S, Stollenwerk B, et al. Cost effectiveness of rivaroxaban for stroke prevention in German patients with atrial fibrillation. *Pharmacoeconomics* 2015;33(3):271-83. doi: 10.1007/s40273-014-0236-9 [published Online First: 2014/11/19]
55. Nguyen E, Egri F, Mearns ES, et al. Cost-Effectiveness of High-Dose Edoxaban Compared with Adjusted-Dose Warfarin for Stroke Prevention in Non-Valvular Atrial Fibrillation Patients. *Pharmacotherapy* 2016;36(5):488-95. doi: 10.1002/phar.1746 [published Online First: 2016/03/27]
56. Rattanachotphanit T, Limwattananon C, Waleekhachonloet O, et al. Cost-Effectiveness Analysis of Direct-Acting Oral Anticoagulants for Stroke Prevention in Thai Patients with Non-Valvular Atrial Fibrillation and a High Risk of Bleeding. *Pharmacoeconomics* 2019;37(2):279-89. doi: 10.1007/s40273-018-0741-3 [published Online First: 2018/11/06]
57. Rognoni C, Marchetti M, Quaglini S, et al. Edoxaban versus warfarin for stroke prevention in non-valvular atrial fibrillation: a cost-effectiveness analysis. *J Thromb Thrombolysis* 2015;39(2):149-54. doi: 10.1007/s11239-014-1104-3 [published Online First: 2014/06/29]
58. Costa J, Fiorentino F, Caldeira D, et al. Cost-effectiveness of non-vitamin K antagonist oral anticoagulants for atrial fibrillation in Portugal. *Revista Portuguesa de Cardiologia* 2015;34(12):723-37. doi: 10.1016/j.repc.2015.07.004 [published Online First: 2015/12/01]
59. Kleintjens J, Li X, Simoens S, et al. Cost-effectiveness of rivaroxaban versus warfarin for stroke prevention in atrial fibrillation in the Belgian healthcare setting. *Pharmacoeconomics* 2013;31(10):909-18. doi: 10.1007/s40273-013-0087-9 [published Online First: 2013/09/14]
60. Sorensen SV, Kansal AR, Connolly S, et al. Cost-effectiveness of dabigatran etexilate for the prevention of stroke and systemic embolism in atrial fibrillation: a Canadian payer perspective. *Thromb Haemost* 2011;105(5):908-19. doi: 10.1160/th11-02-0089 [published Online First: 2011/03/25]
61. Zhao YJ, Lin L, Zhou HJ, et al. Cost-effectiveness modelling of novel oral anticoagulants incorporating real-world elderly patients with atrial fibrillation. *Int J Cardiol* 2016;220:794-801. doi: 10.1016/j.ijcard.2016.06.087 [published Online First: 2016/07/12]
62. Lekuona I, Anguita M, Zamorano JL, et al. Would the Use of Edoxaban Be Cost-effective for the Prevention of Stroke and Systemic Embolism in Patients With Nonvalvular Atrial Fibrillation in Spain? *Rev Esp Cardiol (Engl Ed)* 2019;72(5):398-406. doi: 10.1016/j.rec.2018.03.024 [published Online First: 2019/04/23]
63. Lanas F, Castro C, Vallejos C, et al. Latin American Clinical Epidemiology Network Series - Paper 2: Apixaban was cost-effective vs. acenocoumarol in patients with nonvalvular atrial fibrillation with moderate to severe risk of embolism in Chile. *J Clin Epidemiol* 2017;86:75-83. doi: 10.1016/j.jclinepi.2016.05.018 [published Online First: 2016/10/21]
64. Wu B, Kun L, Liu X, et al. Cost-effectiveness of different strategies for stroke prevention in patients with atrial fibrillation in a health resource-limited setting. *Cardiovasc Drugs Ther* 2014;28(1):87-98. doi: 10.1007/s10557-013-6490-9 [published Online First: 2013/09/21]

65. Langkilde LK, Bergholdt Asmussen M, Overgaard M. Cost-effectiveness of dabigatran etexilate for stroke prevention in non-valvular atrial fibrillation. Applying RE-LY to clinical practice in Denmark. *J Med Econ* 2012;15(4):695-703. doi: 10.3111/13696998.2012.673525 [published Online First: 2012/03/09]
66. Gonzalez-Juanatey JR, Alvarez-Sabin J, Lobos JM, et al. Cost-effectiveness of dabigatran for stroke prevention in non-valvular atrial fibrillation in Spain. *Rev Esp Cardiol (Engl Ed)* 2012;65(10):901-10. doi: 10.1016/j.recesp.2012.06.006 [published Online First: 2012/09/11]
67. Chang CH, Yang YH, Chen JH, et al. Cost-effectiveness of dabigatran etexilate for the prevention of stroke and systemic embolism in atrial fibrillation in Taiwan. *Thromb Res* 2014;133(5):782-9. doi: 10.1016/j.thromres.2014.02.024 [published Online First: 2014/03/20]
68. Kim H, Kim H, Cho SK, et al. Cost-Effectiveness of Rivaroxaban Compared to Warfarin for Stroke Prevention in Atrial Fibrillation. *Korean circulation journal* 2019;49(3):252-63. doi: 10.4070/kcj.2018.0220 [published Online First: 2018/11/24]
69. Vilain KA, Yang MC, Hui Tan EC, et al. Cost-Effectiveness of Edoxaban vs. Warfarin in Patients with Atrial Fibrillation Based on Results of the ENGAGE AF - TIMI 48 Trial: Taiwanese Perspective. *Value Health Reg Issues* 2017;12:74-83. doi: 10.1016/j.vhri.2017.03.011 [published Online First: 2017/06/27]
70. Carles M, Brosa M, Souto JC, et al. Cost-effectiveness analysis of dabigatran and anticoagulation monitoring strategies of vitamin K antagonist. *BMC Health Serv Res* 2015;15:289. doi: 10.1186/s12913-015-0934-9 [published Online First: 2015/07/29]
71. Wang Y, Xie F, Kong MC, et al. Cost-effectiveness of dabigatran and rivaroxaban compared with warfarin for stroke prevention in patients with atrial fibrillation. *Cardiovasc Drugs Ther* 2014;28(6):575-85. doi: 10.1007/s10557-014-6558-1 [published Online First: 2014/10/17]
72. Hospodar AR, Smith KJ, Zhang Y, et al. Comparing the Cost Effectiveness of Non-vitamin K Antagonist Oral Anticoagulants with Well-Managed Warfarin for Stroke Prevention in Atrial Fibrillation Patients at High Risk of Bleeding. *Am J Cardiovasc Drugs* 2018;18(4):317-25. doi: 10.1007/s40256-018-0279-y [published Online First: 2018/05/10]
73. Liu CY, Chen HC. Cost-Effectiveness Analysis of Apixaban, Dabigatran, Rivaroxaban, and Warfarin for Stroke Prevention in Atrial Fibrillation in Taiwan. *Clin Drug Investig* 2017;37(3):285-93. doi: 10.1007/s40261-016-0487-7 [published Online First: 2016/12/19]
74. Lip GY, Lanitis T, Kongnakorn T, et al. Cost-effectiveness of Apixaban Compared With Edoxaban for Stroke Prevention in Nonvalvular Atrial Fibrillation. *Clin Ther* 2015;37(11):2476-88.e27. doi: 10.1016/j.clinthera.2015.09.005 [published Online First: 2015/10/20]
75. van Hulst M, Stevanovic J, Jacobs MS, et al. The cost-effectiveness and monetary benefits of dabigatran in the prevention of arterial thromboembolism for patients with non-valvular atrial fibrillation in the Netherlands. *J Med Econ* 2018;21(1):38-46. doi: 10.1080/13696998.2017.1372222 [published Online First: 2017/08/25]
76. Mendoza JA, Silva FA, Rangel LM. Cost-effectiveness of new oral anticoagulants and warfarin in atrial fibrillation from adverse events perspective. *Revista Colombiana de Cardiologia* 2019;26(2):70-77. doi: 10.1016/j.rccar.2018.10.011
77. Lee S, Anglade MW, Pham D, et al. Cost-effectiveness of rivaroxaban compared to warfarin for stroke prevention in atrial fibrillation. *Am J Cardiol* 2012;110(6):845-51. doi: 10.1016/j.amjcard.2012.05.011 [published Online First: 2012/06/02]
78. Wouters H, Thijs V, Annemans L. Cost-effectiveness of dabigatran etexilate in the prevention of stroke and systemic embolism in patients with atrial fibrillation in Belgium. *J Med Econ* 2013;16(3):407-14. doi: 10.3111/13696998.2013.766200 [published Online First: 2013/01/17]
79. Kourlaba G, Maniadakis N, Andrikopoulos G, et al. Economic evaluation of rivaroxaban in stroke prevention for patients with atrial fibrillation in Greece. *Cost effectiveness and resource allocation : C/E* 2014;12(1):5. doi: 10.1186/1478-7547-12-5 [published Online First: 2014/02/12]

80. Kansal AR, Sorensen SV, Gani R, et al. Cost-effectiveness of dabigatran etexilate for the prevention of stroke and systemic embolism in UK patients with atrial fibrillation. *Heart* 2012;98(7):573-78. doi: 10.1136/heartjnl-2011-300646
81. Kamel H, Johnston SC, Easton JD, et al. Cost-effectiveness of dabigatran compared with warfarin for stroke prevention in patients with atrial fibrillation and prior stroke or transient ischemic attack. *Stroke* 2012;43(3):881-3. doi: 10.1161/strokeaha.111.641027 [published Online First: 2012/02/07]
82. Galvani G, Grassetto A, Sterlicchio S, et al. Cost-Effectiveness of Dabigatran Exilate in Treatment of Atrial Fibrillation. *Journal of atrial fibrillation* 2015;7(6):1223. doi: 10.4022/jafib.1223 [published Online First: 2015/04/30]
83. Hallinen T, Soini EJ, Linna M, et al. Cost-effectiveness of apixaban and warfarin in the prevention of thromboembolic complications among atrial fibrillation patients. *Springerplus* 2016;5(1):1354. doi: 10.1186/s40064-016-3024-5 [published Online First: 2016/09/03]
84. Bergh M, Marais CA, Miller-Janson H, et al. Economic appraisal of dabigatran as first-line therapy for stroke prevention in atrial fibrillation. *S Afr Med J* 2013;103(4):241-5. doi: 10.7196/samj.6471 [published Online First: 2013/04/04]
85. Dwiprahasto I, Kristin E, Endarti D, et al. Cost effectiveness analysis of rivaroxaban compared to warfarin and aspirin for stroke prevention atrial fibrillation (SPAF) in the Indonesian healthcare setting. *Indonesian Journal of Pharmacy* 2019;30(1):74-84. doi: 10.14499/indonesianjpharm30iss1pp74
86. Cowper PA, Sheng S, Lopes RD, et al. Economic Analysis of Apixaban Therapy for Patients With Atrial Fibrillation From a US Perspective: Results From the ARISTOTLE Randomized Clinical Trial. *JAMA Cardiol* 2017;2(5):525-34. doi: 10.1001/jamacardio.2017.0065 [published Online First: 2017/03/30]
87. de Souza CPR, Santoni NB, de Melo TG, et al. Cost-Effectiveness and Cost-Utility Analyses of Dabigatran Compared with Warfarin in Patients with Nonvalvular Atrial Fibrillation and Risk Factors for Stroke and Systemic Embolism within Brazilian Private and Public Health Care Systems Perspectives. *Value Health Reg Issues* 2015;8:36-42. doi: 10.1016/j.vhri.2015.02.003
88. Salcedo J, Hay JW, Lam J. Cost-effectiveness of rivaroxaban versus warfarin for treatment of nonvalvular atrial fibrillation in patients with worsening renal function. *Int J Cardiol* 2019;282:53-58. doi: 10.1016/j.ijcard.2018.11.087 [published Online First: 2018/12/07]
89. Salata BM, Hutton DW, Levine DA, et al. Cost-Effectiveness of Dabigatran (150 mg Twice Daily) and Warfarin in Patients >/= 65 Years With Nonvalvular Atrial Fibrillation. *Am J Cardiol* 2016;117(1):54-60. doi: 10.1016/j.amjcard.2015.09.048 [published Online First: 2015/11/11]
90. Shah SV, Gage BF. Cost-effectiveness of dabigatran for stroke prophylaxis in atrial fibrillation. *Circulation* 2011;123(22):2562-70. doi: 10.1161/circulationaha.110.985655 [published Online First: 2011/05/25]
91. Freeman JV, Zhu RP, Owens DK, et al. Cost-effectiveness of dabigatran compared with warfarin for stroke prevention in atrial fibrillation. *Ann Intern Med* 2011;154(1):1-11. doi: 10.7326/0003-4819-154-1-201101040-00289 [published Online First: 2010/11/03]
92. Chevalier J, Delaitre O, Hammes F, et al. Cost-effectiveness of dabigatran versus vitamin K antagonists for the prevention of stroke in patients with atrial fibrillation: a French payer perspective. *Arch Cardiovasc Dis* 2014;107(6-7):381-90. doi: 10.1016/j.acvd.2014.04.009 [published Online First: 2014/06/29]
93. Nshimyumukiza L, Duplantie J, Gagnon M, et al. Dabigatran versus warfarin under standard or pharmacogenetic-guided management for the prevention of stroke and systemic thromboembolism in patients with atrial fibrillation: a cost/utility analysis using an analytic decision model. *Thrombosis journal* 2013;11(1):14. doi: 10.1186/1477-9560-11-14 [published Online First: 2013/07/20]
94. Hernandez I, Smith KJ, Zhang Y. Cost-effectiveness of non-vitamin K antagonist oral anticoagulants for stroke prevention in patients with atrial fibrillation at high risk of bleeding and normal kidney function. *Thromb Res* 2017;150:123-30. doi: 10.1016/j.thromres.2016.10.006 [published Online First: 2016/10/25]

95. Peng S, Deger KA, Ustyugova A, et al. Cost-effectiveness analysis of dabigatran versus rivaroxaban for stroke prevention in patients with non-valvular atrial fibrillation using real-world evidence in elderly US Medicare beneficiaries. *Curr Med Res Opin* 2018;34(1):55-63. doi: 10.1080/03007995.2017.1375470 [published Online First: 2017/09/02]
96. Clemens A, Peng S, Brand S, et al. Efficacy and cost-effectiveness of dabigatran etexilate versus warfarin in atrial fibrillation in different age subgroups. *Am J Cardiol* 2014;114(6):849-55. doi: 10.1016/j.amjcard.2014.06.015 [published Online First: 2014/08/12]
97. Kansal AR, Sharma M, Bradley-Kennedy C, et al. Dabigatran versus rivaroxaban for the prevention of stroke and systemic embolism in atrial fibrillation in Canada. Comparative efficacy and cost-effectiveness. *Thromb Haemost* 2012;108(4):672-82. doi: 10.1160/th12-06-0388 [published Online First: 2012/08/18]
98. Davidson T, Husberg M, Janzon M, et al. Cost-effectiveness of dabigatran compared with warfarin for patients with atrial fibrillation in Sweden. *Eur Heart J* 2013;34(3):177-83. doi: 10.1093/eurheartj/ehs157 [published Online First: 2012/06/27]
99. Morais J, Aguiar C, McLeod E, et al. Cost-effectiveness of rivaroxaban for stroke prevention in atrial fibrillation in the Portuguese setting. *Revista Portuguesa de Cardiologia* 2014;33(9):535-44. doi: 10.1016/j.repc.2014.02.020 [published Online First: 2014/09/23]
100. Hersi AS, Osenenko KM, Kherraf SA, et al. Cost-effectiveness of apixaban for stroke prevention in non-valvular atrial fibrillation in Saudi Arabia. *Ann Saudi Med* 2019;39(4):265-78. doi: 10.5144/0256-4947.2019.265 [published Online First: 2019/08/06]
101. Krejczy M, Harenberg J, Marx S, et al. Comparison of cost-effectiveness of anticoagulation with dabigatran, rivaroxaban and apixaban in patients with non-valvular atrial fibrillation across countries. *J Thromb Thrombolysis* 2014;37(4):507-23. doi: 10.1007/s11239-013-0989-6 [published Online First: 2013/11/14]
102. Pink J, Pirmohamed M, Hughes DA. Comparative effectiveness of dabigatran, rivaroxaban, apixaban, and warfarin in the management of patients with nonvalvular atrial fibrillation. *Clin Pharmacol Ther* 2013;94(2):269-76. doi: 10.1038/clpt.2013.83
103. Thom HHZ, Hollingworth W, Sofat R, et al. Directly Acting Oral Anticoagulants for the Prevention of Stroke in Atrial Fibrillation in England and Wales: Cost-Effectiveness Model and Value of Information Analysis. *MDM policy & practice* 2019;4(2):2381468319866828. doi: 10.1177/2381468319866828 [published Online First: 2019/08/28]
104. You JHS. Novel oral anticoagulants versus warfarin therapy at various levels of anticoagulation control in atrial fibrillation - A cost-effectiveness analysis. *J Gen Intern Med* 2014;29(3):438-46. doi: 10.1007/s11606-013-2639-2
105. Chen YF, Han HN. Economic evaluation of dabigatran, rivaroxaban and warfarin in preventing stroke in patients with atrial fibrillation. *Chinese Journal of New Drugs* 2016;25(11):1216-24.
106. Canal Fontcuberta C, Betegón Nicolás L, Escolar Albaladejo G, et al. Cost-effectiveness analysis of apixaban versus rivaroxaban in the prevention of stroke in patients with non-valvular atrial fibrillation in Spain. *Pharmacoeconomics - Spanish Research Articles* 2015;12(3):93-103. doi: 10.1007/s40277-015-0041-7
107. García-Peña ÁA. Cost-effectiveness assessment of new oral anticoagulation drugs in patients with non-valvular atrial fibrillation. *Revista Colombiana de Cardiologia* 2017;24(2):87-95. doi: 10.1016/j.rccar.2016.07.012
108. Silva Miguel L, Ferreira J. Clinical and economic consequences of using dabigatran or rivaroxaban in patients with non-valvular atrial fibrillation. *Revista Portuguesa de Cardiologia* 2016;35(3):141-48. doi: 10.1016/j.repc.2015.09.009
109. Miguel LS, Rocha E, Ferreira J. Economic evaluation of dabigatran for stroke prevention in patients with non-valvular atrial fibrillation. *Revista Portuguesa de Cardiologia* 2013;32(7-8):557-65. doi: 10.1016/j.repc.2013.01.005
110. Ravasio R, Pedone MP, Ratti M. Cost efficacy analysis of new oral anticoagulant for stroke prevention in non-valvular atrial fibrillation in Italy. *PharmacoEconomics - Italian Research Articles* 2014;16(2-3):1-10. doi: 10.1007/s40276-014-0022-x

111. Triana JJ, Castañeda C, Parada L, et al. Cost-effectiveness of dabigatran compared with warfarin in the treatment of patients with non valvular atrial fibrillation in Colombia. *Revista Colombiana de Cardiologia* 2016;23(2):82-86. doi: 10.1016/j.rccar.2015.06.010
112. Rudakova AV, Parfenov VA. Cost-Effectiveness Of Apixaban Compared To Warfarin And Aspirin In Patients With Non-Valvular Atrial Fibrillation (Nvaf) In The Russian Federation. *Value Health* 2014;17(7):A489. doi: 10.1016/j.jval.2014.08.1441 [published Online First: 2014/11/01]
113. Oyaguez I, Suarez C, Lopez-Sendon JL, et al. Cost-Effectiveness Analysis of Apixaban Versus Edoxaban in Patients with Atrial Fibrillation for Stroke Prevention. *PharmacoEconomics - open* 2019 doi: 10.1007/s41669-019-00186-7 [published Online First: 2019/11/02]
114. Hori M, Tanahashi N, Akiyama S, et al. Cost-effectiveness of rivaroxaban versus warfarin for stroke prevention in non-valvular atrial fibrillation in the Japanese healthcare setting. *J Med Econ* 2019:1-10. doi: 10.1080/13696998.2019.1688821 [published Online First: 2019/11/07]
115. Ng SS, Nathisuwan S, Phrommintikul A, et al. Cost-effectiveness of warfarin care bundles and novel oral anticoagulants for stroke prevention in patients with atrial fibrillation in Thailand. *Thromb Res* 2020;185:63-71. doi: 10.1016/j.thromres.2019.11.012 [published Online First: 2019/11/27]
116. de Jong LA, Groeneveld J, Stevanovic J, et al. Cost-effectiveness of apixaban compared to other anticoagulants in patients with atrial fibrillation in the real-world and trial settings. *PLoS One* 2019;14(9):e0222658. doi: 10.1371/journal.pone.0222658 [published Online First: 2019/09/19]
117. de Pouvourville G, Blin P, Karam P. The contribution of real-world evidence to cost-effectiveness analysis: case study of Dabigatran etexilate in France. *Eur J Health Econ* 2019 doi: 10.1007/s10198-019-01123-5 [published Online First: 2019/10/28]
118. Táborský M, Tomek A, Cihák R, et al. Cost-efectiveness analysis of fi rst-line NOAC prevention of stroke and systemic embolism in patients with non-valvular atrial fi brillation. *Cor et Vasa* 2019;61(4):354-69. doi: 10.33678/cor.2019.058

Noviyani R, *et al. BMJ EBM* 2022; 27:215–223. doi: 10.1136/bmjebm-2020-111634
